# A novel urinary biomarker predicts 1-year mortality after discharge from intensive care

**DOI:** 10.1186/s13054-019-2686-0

**Published:** 2020-01-09

**Authors:** Esther Nkuipou-Kenfack, Agnieszka Latosinska, Wen-Yi Yang, Marie-Céline Fournier, Alice Blet, Blerim Mujaj, Lutgarde Thijs, Elodie Feliot, Etienne Gayat, Harald Mischak, Jan A. Staessen, Alexandre Mebazaa, Zhen-Yu Zhang, The French and European Outcome Registry in Intensive Care Unit Investigators, N. Deye, C. Fauvaux, A. Mebazaa, C. Damoisel, D. Payen, E. Azoulay, A. S. Moreau, L. Jacob, O. Marie, M. Wolf, R. Sonneville, R. Bronchard, I. Rennuit, C. Paugam, J. P. Mira, A. Cariou, A. Tesnières, N. Dufour, N. Anguel, L. Guérin, J. Duranteau, C. Ract, M. Leone, B. Pastène, T. Sharshar, A. Fayssoyl, J. L. Baudel, B. Guidet, Q. Lu, W. Jie Gu, N. Brechot, A. Combes, S. Jaber, A. Pradel, Y. Coisel, M. Conseil, A. Vieillard-Baron, L. Bodson, J. Y. Lefrant, L. Elotmani, A. Ayral, S. Lloret, S. Pily-Flouri, J. B. Pretalli, P. F. Laterre, V. Montiel, M. F. Dujardin, C. Berghe

**Affiliations:** 1 Mosaiques Diagnostics GmbH, Hannover, Germany; 2 Studies Coordinating Centre, Research Unit Hypertension and Cardiovascular Epidemiology, KU Leuven Department of Cardiovascular Sciences, University of Leuven, Campus Sint Rafaël, Kapucijnenvoer 35, Box 7001, 3000 Leuven, Belgium; 3 Department of Cardiology, Shanghai General Hospital, Shanghai Jiao Tong University School of Medicine, Shanghai, China; 4 Department of Anesthesiology and Intensive Care, Saint Louis-Lariboisière – Fernand Widal University Hospital, Assistance Publique Hôpitaux de Paris, Paris, France; 5 Université de Paris, Paris, France; 6 INSERM UMR-S 942 – MASCOT, Paris, France; 7 Cardiovascular Research Institute Maastricht, Maastricht University, Maastricht, the Netherlands

**Keywords:** Biomarker, Intensive care medicine, Heart failure, Mortality, Urinary proteomics

## Abstract

**Rationale:**

The urinary proteome reflects molecular drivers of disease.

**Objectives:**

To construct a urinary proteomic biomarker predicting 1-year post-ICU mortality.

**Methods:**

In 1243 patients, the urinary proteome was measured on ICU admission, using capillary electrophoresis coupled with mass spectrometry along with clinical variables, circulating biomarkers (BNP, hsTnT, active ADM, and NGAL), and urinary albumin. Methods included support vector modeling to construct the classifier, Cox regression, the integrated discrimination (IDI), and net reclassification (NRI) improvement, and area under the curve (AUC) to assess predictive accuracy, and Proteasix and protein-proteome interactome analyses.

**Measurements and main results:**

In the discovery (deaths/survivors, 70/299) and test (175/699) datasets, the new classifier ACM128, mainly consisting of collagen fragments, yielding AUCs of 0.755 (95% CI, 0.708–0.798) and 0.688 (0.656–0.719), respectively. While accounting for study site and clinical risk factors, hazard ratios in 1243 patients were 2.41 (2.00–2.91) for ACM128 (+ 1 SD), 1.24 (1.16–1.32) for the Charlson Comorbidity Index (+ 1 point), and ≥ 1.19 (*P* ≤ 0.022) for other biomarkers (+ 1 SD). ACM128 improved (*P* ≤ 0.0001) IDI (≥ + 0.50), NRI (≥ + 53.7), and AUC (≥ + 0.037) over and beyond clinical risk indicators and other biomarkers. Interactome mapping, using parental proteins derived from sequenced peptides included in ACM128 and in silico predicted proteases, including/excluding urinary collagen fragments (63/35 peptides), revealed as top molecular pathways protein digestion and absorption, lysosomal activity, and apoptosis.

**Conclusions:**

The urinary proteomic classifier ACM128 predicts the 1-year post-ICU mortality over and beyond clinical risk factors and other biomarkers and revealed molecular pathways potentially contributing to a fatal outcome.

## Introduction

In high- and middle-income countries, millions of patients survive critical illness thanks to the highly specialized life-sustaining management in intensive care units (ICU). However, cumulative mortality over the first year after ICU discharge ranges from 26 to 63% [1]. Large cohort studies conducted in Canada [2], Australia [3], and the USA [1] demonstrated that ICU survivors followed up from 3 [1, 2] up to 15 [3] years experienced mortality rates 2 to 5 times higher than sex- and age-matched population controls. The number of patients who survive intensive care is growing fast, because of the demographic transition in aging populations [4] and the ongoing sophistication of critical care resulting in a lower in-ICU fatality rate [5–7]. Several risk factors determine the 1-year risk of death after ICU discharge. Clinical risk indicators include older age, the indication for critical care, comorbidities, the number of failing organs, the length of ICU care, and newly diagnosed malignancies [3]. The risk of death is also associated with circulating and urinary biomarkers indicative of myocardial, vascular, or renal distress [8]. Stakeholder conferences advised prioritizing research on reliable predictors of post-ICU impairments and death to identify patients in need of further diagnostic work-up and targeted treatment [5, 7]. Urinary proteomic profiling developed over the past 15 years into a state-of-the-art technology, which enables discovery of disease-specific multidimensional biomarkers indicative of molecular pathogenic processes [9, 10]. Along these lines, the current study aimed to develop a urinary proteomic classifier predictive of the 1-year mortality in ICU survivors. The French and European Outcome Registry in Intensive Care Unit Investigators (FROG-ICU; (NCT01367093) compiled the analyzed database [8, 11].

## Methods

### Patients

FROG-ICU involved medical, surgical, or mixed ICUs at 15 university hospitals [11]. Inclusion criteria included mechanical ventilation or administration of vasoactive agents for at least 24 h. The exclusion criteria were age less than 18 years, severe head injury with a Glasgow Coma Scale [12] below 8, brain death or persistent vegetative state, pregnancy or breastfeeding, transplantation in the past 12 months, moribund status, and lack of social security coverage [11].

### Measurements

Anthropometric, clinical, and routine biochemical data were recorded on ICU admission. Variables of interest included the indication for admission to the ICU, the Charlson Comorbidity Index (CCI) [13, 14], the Sequential Organ Failure Assessment (SOFA) [15] score, blood pressure, serum creatinine and cystatin C, and blood glucose. Information also collected on admission, included treatment with mechanical ventilation, extracorporeal membrane oxygenation, or renal replacement therapy.

The CCI is a method of categorizing comorbidities of patients based on the International Classification of Diseases (ICD) diagnosis codes found in administrative data. It included 19 categories (Additional file 1: Table S1). Each comorbidity category has an associated weight (from 1 to 6), based on the adjusted risk of mortality or resource use, and the sum of all the weights results in a single comorbidity score for a patient. A CCI was calculated for each patient on ICU admission to classify comorbidity and grouped as having either no comorbidity (CCI = 0), moderate comorbidity (CCI = 1–5), or severe comorbidity (CCI ≥ 6). SOFA score can help assess the degree of organ dysfunction on ICU admission. The score is based on six different scores, one each for the respiratory, cardiovascular, hepatic, coagulation, renal, and neurological systems. If an organ is not affected, a zero score is given. If an organ system is affected in multiple ways, the highest score is used in constructing the SOFA categorization. SOFA scores range from 0 to 24, and the risk of death is proportional to the score.

Estimated glomerular filtration rate was derived from serum creatinine (eGFR_crt_) or cystatin C (eGFR_cys_), according to the Chronic Kidney Disease Epidemiology Collaboration equation [16]. Fresh urine samples were analyzed for the albumin concentration.

### Biomarkers

Circulating and urinary biomarkers were measured in a central certified laboratory on samples obtained on ICU admission. The circulating biomarkers indicated left ventricular dysfunction (brain natriuretic peptide [BNP]; Roche Diagnostics GmbH, Mannheim, Germany), left ventricular ischemia and injury (high-sensitive troponin T [hsTNT]; Abbott, Abbott Park, IL) [17], myocardial ischemia (high-sensitive troponin I [hsTnI]; Abbott, Abbott Park, IL) [18], vascular dysfunction (biologically active adrenomedullin [ADM]; Adrenomed GmbH, Hennigsdorf, Germany) [19], inflammation and cardiac stress (the interleukin-1 receptor family member soluble ST2 [sST2]; Eurobio, Critical Diagnostics, San Diego, CA) [20], or acute kidney injury (neutrophil-gelatinase-associated lipocalin [NGAL]; ARCHITECT, Abbott Diagnostics, Chicago, IL) [21]. The urinary biomarkers included albuminuria and NGAL [21].

The urinary proteome is well characterized, and reference standards are available [22]. Urine proteome analysis was performed on urine samples collected on admission and bio-banked until assayed. Detailed information on urine sample preparation, proteome analysis by capillary electrophoresis coupled to mass spectrometry, data processing, and sequencing of the urinary peptides allowing identification of parent proteins is available in previous publications [9] and the methods section of the online-only supplement.

### Outcome

Information on vital status was collected 3, 6, and 12 months after ICU discharge, as previously described [11]. Trained clinical research assistants called the patients or their relatives. The short intervals between contacts, ranging from 3 to 6 months, established a strong relationship of trust between the research team, the patients, and their family. If this direct contact was lost during follow-up, vital status was ascertained via national health services records. For the current study, the 1-year vital status was known for all patients included in the analysis.

### Statistics

SAS, version 9.4, maintenance level 5 (SAS Institute Inc., Cary, NC) was used for database management and statistical analysis. Departure of distributions from normality was evaluated by the Shapiro-Wilk test. The biomarker distributions with the exception of ACM128 were transformed by sorting measurements from the smallest to the highest and then applying the inverse cumulative normal function [23]. Means, medians, and proportions were compared using the large-sample *z*-test or ANOVA, the Wilcoxon rank-sum test, and Fisher’s exact test, respectively. We computed 95% confidence interval of rates as $R\pm 1.96\times \sqrt{\left(R/T\right)}$, where *R* and *T* are the rate and the denominator used to calculate the rate.

#### Construction and initial validation of the classifier

For discovery of the new classifier associated with all-cause mortality following ICU discharge, 30% of the cohort (299 survivors and 70 nonsurvivors) were randomly selected from all enrolled patients (*n* = 1243). For validation, the remainder of the cohort was analyzed (699 survivors and 175 nonsurvivors). In the discovery phase, the association of mortality with urinary peptides was assessed by Wilcoxon testing targeting a significance level of 0.05. The peptides that remained associated with the 1-year mortality were combined into a single multidimensional classifier, using support vector machine modeling, as implemented in the MosaCluster software, version 1.7.0, and as described in detail in the methods section of the online supplement.

#### Performance of the classifier

In patients included in the validation data set, performance of the new classifier was assessed, using proportional hazards regression. The comparators were established clinical predictors of adverse health outcomes in ICU patients, i.e., the Charlson Comorbidity Index [13, 14] and SOFA [15] score and other circulating and urinary biomarkers. These analyses accounted for center effects as a random variable and for risk factors as fixed effects. Centers were grouped per location (*n* = 15) or per group of patients they were serving (medical, surgical, or mixed). First, cumulative incidence of death was plotted by thirds of the distribution of the new classifier, while accounting for sex, age, and Charlson Comorbidity Index. Next, the hazard ratios relating the risk of death to tertiles of classifier were computed from Cox models adjusted for sex, age, mean arterial pressure (diastolic pressure plus one third of pulse pressure), eGFR, and diabetes mellitus. The proportional hazards assumption was checked by the Kolmogorov-type supremum test and by testing the interaction between follow-up duration and the new classifier. Finally, in multivariable-adjusted Cox models, the accuracy of the classifier to discriminate between survivors and nonsurvivors was compared with the Charlson Comorbidity Index [13] and other biomarkers (one at a time). Improvement in model performance was assessed from the integrated discrimination improvement (IDI) [24], the net reclassification improvement (NRI) [24], and the change in the area under the curve (∆UC).

#### Analysis of single urinary peptides

For analysis of single peptides, sequenced peptides, which had a detectable signal in over 70% of participants, were selected. *P* values and confidence intervals were adjusted for multiple testing using the Bonferroni method based on the number of parental proteins identified.

#### Proteasix and pathway analysis

Proteases responsible for the generation of the urinary peptide fragments were predicted in silico, using Proteasix [25, 26]. Proteolytic enzymes mapped to at least 10 cleavage sites in the peptides associated with post-ICU mortality were analyzed. The protein-protein interactome was constructed, using the STRING database (STRING, version 11.0; https://string-db.org). Proteins corresponding to sequenced urinary peptides and the in silico predicted proteolytic enzymes were included for the interactome analysis. The pathway enrichment was evaluated against the Kyoto Encyclopedia of Genes and Genomes (KEGG) database with the false discovery rate set at *P* < 0.01.

Changes in urinary collagen fragments might reflect alterations in protease activity, but may also be the result of a change in collagen turnover in tissues, such as for instance as a consequence of increased cross-linking. Imputation of the protease activity based on directional changes in urinary collagen fragments might be incorrect. To account for this potential problem, we also performed a Proteasix analysis, excluding collagen fragments.

## Results

### Characteristics of patients

The analysis included 998 survivors and 245 nonsurvivors. Nonsurvivors compared with survivors (Table 1) included more patients with diabetes mellitus (26.5% vs. 14.5%) and needing dialysis (10.2 vs. 5.7%). Nonsurvivors were older (68.9 vs. 57.2 years), had lower diastolic blood pressure (61.7 vs. 64.5 mmHg), mean arterial pressure (82.6 vs. 84.8 mmHg), lower eGFR_crt_ (80.1 vs. 98.8 mL/min/1.73 m^2^) and eGFR_cys_ (48.3 vs. 75.8 mL/min/1.73 m^2^), but higher Charlson Comorbidity Index (5 vs. 2 points; Table 1).

**Table 1:** Clinical characteristics at baseline by 1-year survival status

Characteristic	Survivors (*n* = 998)	Nonsurvivors (*n* = 245)	*P* value
*N* with characteristic (%)			
Women	357 (35.8)	84 (35.1)	0.84
Diabetes mellitus	145 (14.5)	65 (26.5)	< 0.0001
Indication of intensive care			
Acute respiratory insufficiency	198 (19.8)	63 (25.7)	0.043
Pancreatitis or liver failure	14 (1.4)	8 (3.3)	0.048
Hemorrhagic or hypovolemic shock	56 (5.6)	21 (8.6)	0.085
Cardiogenic shock or heart failure	145 (14.5)	34 (13.9)	0.79
Sepsis or anaphylactic shock	230 (23.1)	68 (27.8)	0.12
Post-surgical care	101 (10.1)	23 (9.4)	0.73
Severe trauma	78 (7.8)	0	< 0.0001
Other indications	176 (17.6)	28 (11.4)	0.019
Treatment administered			
Mechanical ventilation	615 (61.6)	139 (56.7)	0.16
Extracorporeal membrane oxygenation	9 (0.9)	1 (0.4)	0.44
Dialysis	57 (5.7)	25 (10.2)	0.011
Mean characteristic (SD)			
Age, years	57.2 (16.9)	68.9 (12.3)	< 0.0001
Body mass index, kg/m^2^	27.7 (7.6)	26.8 (5.4)	0.081
Systolic pressure, mmHg	125.7 (22.5)	124.5 (22.6)	0.47
Diastolic pressure, mmHg	64.5 (14.0)	61.7 (13.1)	0.0046
Mean arterial pressure, mmHg	84.8 (14.7)	82.6 (13.6)	0.030
Heart rate, beats per minute	92.5 (20.7)	89.4 (19.7)	0.033
Blood glucose, mmol/L	7.35 (2.33)	7.65 (2.70)	0.22
eGFR_crt_, mL/min/1.73 m^2^	98.8 (55.6)	80.1 (57.9)	< 0.0001
eGFR_cys_, mL/min/1.73 m^2^	75.8 (45.9)	48.3 (36.2)	< 0.0001
Median characteristic (IQR)			
Charlson Comorbidity Index	2 (1–4)	5 (3–6)	< 0.0001
SOFA score	7 (4–10)	8 (5–10)	0.46
Length of ICU stay, days	11 (7–18)	12 (7–22)	0.092

Baseline refers to the date of ICU admission. Body mass index was body weight in kilograms divided by height in meters squared. Mean arterial pressure is diastolic pressure plus one third of the difference between systolic and diastolic pressure. Diabetes mellitus was a fasting/random glucose ≥ 7.0/11.1 mmol/L, use of anti-diabetic agents or a diagnosis in medical records. *P* denotes the significance of the difference between survivors and nonsurvivors

*Abbreviations: eGFR*_*crt*_*/eGFR*_*cys*_ glomerular filtration rate derived from serum creatinine/cystatin C according to the Chronic Kidney Disease Epidemiology Collaboration equation (16), *ICU* intensive care unit, *SOFA* Sequential Organ Failure Assessment Score (15), *IQR* interquartile range

### Discovery and validation

The characteristics of surviving and nonsurviving patients in the discovery and validation datasets were broadly similar (see Additional file 1: Tables S2 and S3 and Figure S1). The analysis of the discovery dataset (number of deaths/survivors, 70/299) enabled identification of 128 peptides, which were significantly associated with the 1-year post-ICU mortality. Of 63 peptides with sequence information available, 28 (44.4%) were collagen fragments (Additional file 1: Table S5). The 128 peptides were combined in a multidimensional classifier, termed ACM128 (Additional file 1: Figure S1). With the C and gamma parameters set at 10,240 and 0.0000002, respectively, the AUC was 0.755 (95% confidence interval [CI], 0.708 to 0.798; *P* < 0.0001) after complete take-one-out cross-validation. The unadjusted hazard ratio for a 1-SD increment in ACM128 was 4.55 (CI, 3.47–5.96; *P* < 0.0001). In the test set (number of deaths/survivors, 175/699), the AUC was 0.688 (CI, 0.656 to 0.719; *P* < 0.0001) and the hazard ratio 2.27 (CI, 1.86–2.78; *P* < 0.0001).

With the exception of urinary albumin (*P* = 0.69), all circulating (BNP, hsTnT, hsTnI, ADM, sSD2, and NGAL) and urinary biomarkers (NGAL and ACM128) had higher levels in nonsurvivors than survivors (Table 2). Most circulating and urinary biomarkers were correlated (see Additional file 1: Table S2). For ACM128, the correlation coefficients were not significant with urinary albumin (*r* = 0.038; *P* = 0.18); the correlation coefficients of ACM128 with the other circulating and urinary biomarkers ranged from 0.19 (*P* = 0.0083) for hsTnI and sST2 to 0.45 (*P* < 0.0001) for plasma NGAL.

**Table 2:** Biomarkers at baseline by 1-year survival status

Biomarkers	Survivors (*n* = 998)	Nonsurvivors (*n* = 245)	*P*
	Median (IQR)	Median (IQR)	
Circulating			
BNP, pg/mL	106 (34, 302)	251 (98, 763)	< 0.0001
hsTNT, pg/L	32 (12, 90)	44 (19, 177)	< 0.0001
hsTnI, pg/L	28 (8, 184)	41 (13, 390)	0.0006
ADM, nmol/L	50 (29, 92)	82 (42, 143)	< 0.0001
sST2, ng/mL	281 (163, 501)	376 (205, 848)	0.0002
NGAL, mg/mL	148 (81, 304)	272 (128, 528)	< 0.0001
Urinary			
Albuminuria, mg/L	381 (300, 467)	386 (293, 461)	0.69
NGAL, ng/mL	84 (31, 390)	200 (46, 902)	< 0.0001
ACM128	− 0.53 (− 0.89, − 0.07)	0.11 (− 0.34, 0.69)	< 0.0001

Values are medians (interquartile range). *P* denotes the significance of the difference between survivors and nonsurvivors

*Abbreviations: BNP* brain natriuretic peptide, *hsTnT* high-sensitive troponin T, *hsTnI* high-sensitive troponin I, *ADM* biologically active adrenomedullin, *sST2* soluble ST2, *NGAL* neutrophil-gelatinase-associated lipocalin, *ACM128* multidimensional urinary proteomic biomarker

### Improvement of model performance

**Fig. 1:**
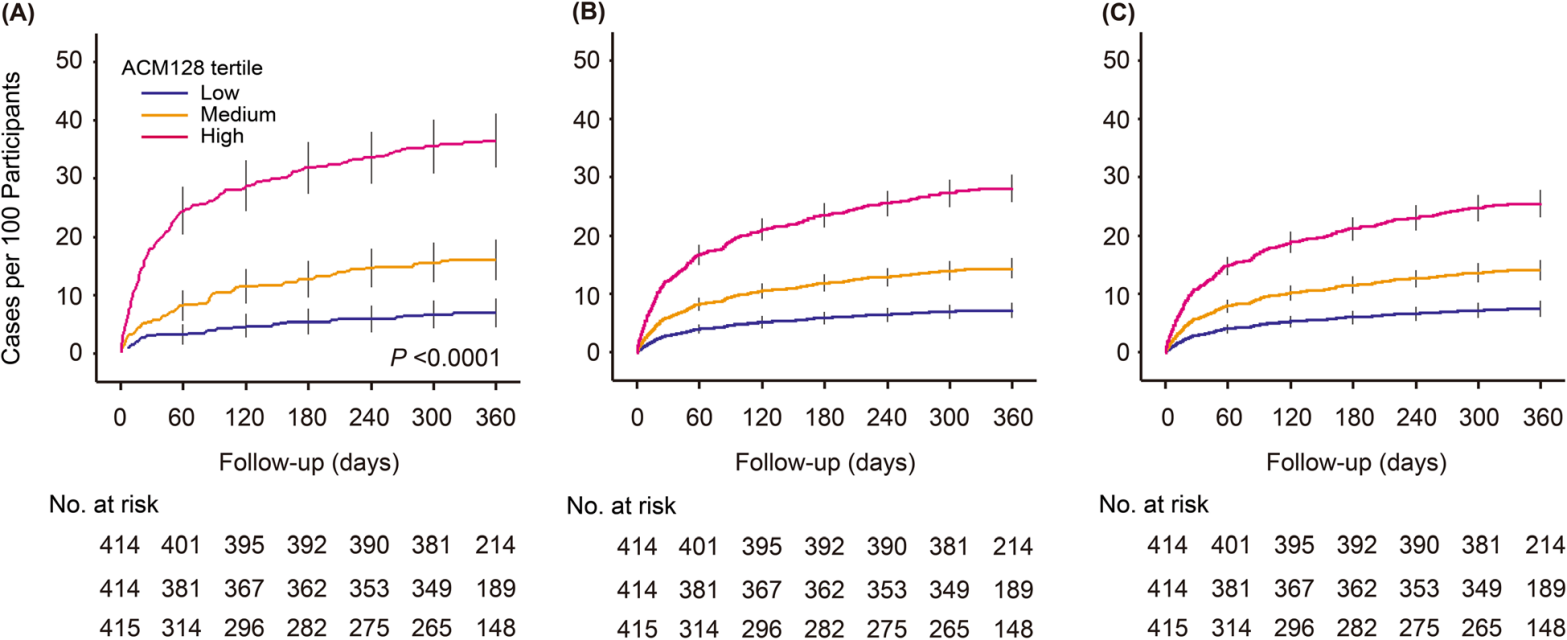
Cumulative incidence of mortality over 1 year in 1243 study participants by thirds of the ACM128 distribution, unadjusted (**a**), adjusted for sex and age (**b**), and additionally adjusted for Charlson Comorbidity Index (**c**). *P* values denote the significance of the difference between the low (≤ − 0.662) and top (> − 0.091) thirds of the ACM128 distribution. The numbers along the horizontal axis denote the number of patients at risk at 60-day intervals

In the whole study sample (*n* = 1243), 245 deaths occurred within 1 year after ICU discharge, yielding a death rate of 19.7 per 100 patient-years (CI, 19.5–19.9 patient-years). With adjustments applied for sex and age, the cumulative death rate increased across thirds of the ACM128 distribution (low vs. top third, 6.7 [CI, 6.4–7.0] vs. 36.2 [CI, 35.6–36.8] deaths per 100 patient-years). Further adjustment for the Charlson Comorbidity Index did not remove this gradient (Fig. 1).

The proportional hazard assumption was met. While adjusting for center (*n* = 15), sex, age, mean arterial pressure, eGFR_cys_, and diabetes mellitus (Table 3), the hazard ratio relating the risk of death to ACM128 (+ 1 SD) was 2.41 (CI, 2.00–2.91). With similar adjustments applied, the hazard ratio for the Charlson Comorbidity Index (+ 1 point) was 1.24 (CI, 1.16–1.32) and for urinary albumin (+ 1 SD) 1.23 (CI, 1.07–1.42). The hazard ratios for the circulating biomarkers (+ 1 SD) ranged from 1.09 (CI, 0.94–1.25) for sST2 to 1.39 (CI, 1.11–1.48) for ADM (Table 3). Substituting eGFR_cys_ as covariable by eGFR_crt_ or replacing the center adjustment by type of ICU ward (medical, surgical, or mixed) yielded highly consistent estimates of the hazard ratios.

**Table 3:** Hazard ratios expressing the risk of death in relation to risk factors and biomarkers in 1243 ICU survivors

Risk factors	Hazard ratio (95% CI)	*P*
Number of deaths/number at risk	245/1243	
Clinical risk factors		
Mechanical ventilation (0, 1)	0.84 (0.65, 1.09)	0.20
Sepsis (0, 1)	0.99 (0.74, 1.32)	0.92
Charlson Comorbidity Index (+ 1 point)	1.24 (1.16, 1.32)	< 0.0001
SOFA score (+ 1 point)	0.99 (0.86, 1.15)	0.92
Length of ICU stay (+ 1 day)	1.18 (1.03, 1.34)	0.014
Circulating biomarkers		
BNP (+ 1 SD)	1.38 (1.19, 1.60)	< 0.0001
hsTNT (+ 1 SD)	1.19 (1.03, 1.39)	0.022
hsTnI (+ 1 SD)	1.14 (0.99, 1.32)	0.076
ADM (+ 1 SD)	1.39 (1.11, 1.48)	0.0006
sST2 (+ 1 SD)	1.09 (0.94, 1.25)	0.23
NGAL (+ 1 SD)	1.28 (1.11, 1.47)	0.0005
Urinary biomarkers		
Albuminuria (+ 1 SD)	1.23 (1.07, 1.42)	0.0046
NGAL (+ 1 SD)	1.13 (0.98, 1.31)	0.094
ACM128 (+ 1 SD)	2.41 (2.00, 2.91)	< 0.0001

Abbreviations of the biomarkers are given in Table 2. All models accounted for center (*n* = 15) as random effect and for sex, age, mean arterial pressure, glomerular filtration estimated from serum cystatin C, and diabetes mellitus

Based on the results presented in Table 3, we carried the Charlson Comorbidity Index, the circulating biomarkers (except hsTnI and sST2), and urinary albumin through to further analyses (Table 4). We assessed model performance based on NRI, IDI, and ∆AUC by adding ACM128 to a model accounting for center, the covariables, and including either the Charlson Comorbidity Index or a second biomarker (Table 5). Adding ADM128 to these models consistently (*P* ≤ 0.0001) increased NRI, NDI, and the AUC (Table 5). Replacing ACM128 by the SOFA score combined with the length of the ICU stay as an index of frailty did not increase the AUC (AUC, 0.74; *P* = 0.31).

**Table 4:** Hazard ratios expressing the risk of death in models adding ACM128 to an established risk factor or biomarker

Risk factor to which ACM128 was added	Estimates for the risk factor to which ACM128 was added		Estimates for ACM128 in models with a second risk factor added	
	HR (95% CI)	*P*	HR (95% CI)	*P*
Clinical risk factors				
Charlson Comorbidity Index	1.20 (1.12, 1.28)	< 0.0001	2.24 (1.86, 2.71)	< 0.0001
Length of ICU stay	1.12 (0.99, 1.27)	0.078	2.38 (1.97, 2.87)	< 0.0001
Circulating biomarkers				
BNP	1.15 (0.99, 1.34)	0.070	2.28 (1.87, 2.77)	< 0.0001
hsTnT	1.03 (0.87, 1.20)	0.76	2.40 (1.98, 2.91)	< 0.0001
ADM	1.06 (0.91, 1.23)	0.46	2.35 (1.93, 2.87)	< 0.0001
NGAL	1.04 (0.89, 1.21)	0.65	2.37 (1.93, 2.90)	< 0.0001
Urinary biomarkers				
Albumin	1.14 (0.99, 1.32)	0.078	2.37 (1.96, 2.86)	< 0.0001

Abbreviations of the biomarkers are given in Table 2. The analysis includes 245 deaths and 1243 patients at risk. All models accounted for center (*n* = 15) as random effect and for sex, age, mean arterial pressure, glomerular filtration estimated from serum cystatin C, and diabetes mellitus. Association sizes are expressed for a 1-SD increment in the biomarkers except for the Charlson Comorbidity Index (+ 1 point)

**Table 5:** Improvement of model performance for adding ACM128 to an established risk factor or biomarker in 1243 ICU survivors

Risk factor to which ACM128 was added	IDI (95% CI)	NRI (95% CI)	AUC of basic model (95% CI)	Increase in AUC by adding ACM128
Clinical risk factors				
Charlson Comorbidity Index	1.12 (0.72, 1.51)	49.3 (35.6, 63.0)	0.746 (0.713, 0.779)	0.042 (0.022, 0.063)
ICU stay	1.24 (0.87, 1.62)	51.3 (37.6, 65.0)	0.719 (0.686, 0.753)	0.057 (0.033, 0.081)
Circulating biomarkers				
BNP	1.12 (0.76, 1.47)	41.0 (27.2, 54.8)	0.726 (0.693, 0.759)	0.048 (0.026, 0.069)
hsTnT	1.23 (0.85, 1.60)	47.9 (34.1, 61.6)	0.718 (0.684, 0.752)	0.056 (0.032, 0.080)
ADM	1.14 (0.78, 1.50)	52.8 (39.2, 66.4)	0.724 (0.691, 0.757)	0.049 (0.028, 0.071)
NGAL	1.11 (0.75, 1.71)	52.8 (39.1, 66.4)	0.729 (0.696, 0.763)	0.045 (0.023, 0.066)
Urinary albumin	1.22 (0.84, 1.60)	46.4 (32.7, 60.1)	0.721 (0.686, 0.755)	0.053 (0.029, 0.077)

Abbreviations of the biomarkers are given in Table 2. The analysis includes 245 deaths and 1243 patients at risk. IDN indicates the integrated discrimination improvement, NRI the net reclassification improvement, and AUC to the area under the curve. All estimates, given with 95% confidence interval, were significant (*P* ≤ 0.0001). All models accounted for center (*n* = 15) as random effect and for sex, age, mean arterial pressure, estimated glomerular filtration estimated from serum cystatin C, and diabetes mellitus. The basic model included the covariables and the risk factor or biomarker to which ACM128 was added. IDI is the difference between the discrimination slopes of the basic model and the basic model extended with ACM128. The discrimination slope is the difference in predicted probabilities (%) between cases and controls. NRI is the sum of the percent of patients reclassified correctly as cases and controls

### Single urinary peptides

The analysis of 153 single urinary peptides with known amino-acid sequence and detectable in over 70% of patients enabled identification of 19 peptides, which were associated with the risk of death with adjustments applied for sex, age, mean arterial pressure, eGFR_cys_, and diabetes mellitus and with significance levels corrected for multiple testing (Additional file 1: Table S7). The risk of death, expressed per 1-SD increment in the marker signal amplitude increased with 14 collagen alpha-1 (I) fragments (1.20 ≤ HR ≤ 1.50; 0.0001 ≤ *P* ≤ 0.0050), three collagen alpha-1 (III) fragments (1.19 ≤ HR ≤ 1.28; 0.00017 ≤ *P* ≤ 0.0049), and one collagen alpha-1 (V) chain (HR, 1.29; *P* = 0.00014). The risk of death decreased with a fibrinogen alpha chain fragment (HR, 0.80; *P* = 0.0013).

### Protease activity and pathway analysis

The in silico analysis of sequenced urinary peptide fragments, including (*n* = 63) and excluding (*n* = 28) collagen, is presented in Additional file 1: Tables S6 and S7, respectively. With collagen fragments included, the proteases with a nonsurvivors-to-survivor ratio of 1.5 or greater included cathepsin E (1.98), D (1.89), G (1.70), S (1.52), and L1 (1.51), kallikrein-6 (1.77), and neutrophil elastase (1.67). The analysis with 28 collagen fragments excluded did not identify kallikrein-6, but was otherwise confirmatory, and additionally predicted matrix metalloproteinase 7 (1.51), 9 (1.61), 14 (1.90), and 25 (1.89), cathepsin B (1.82), S (1.79), and K (1.60), and meprin A subunit alpha (1.61).

The protein-protein interactome derived from 30 parental proteins identified from 63 sequenced urinary peptides included in the ACM128 classifier and the in silico predicted proteases (Additional file 1: Table S7) generated a network consisting of 57 nodes and 295 edges (Additional file 1: Figure S2), with a protein-protein interaction enrichment *P* of 10–16. Excluding the 28 urinary collagen fragment from the Proteasix (Additional file 1: Table S8) and interactome analysis yielded a network of 37 nodes and 122 edges (Additional file 1: Figure S3) with an enrichment *P* of 10–16. Both analyses revealed as top molecular pathways protein digestion and absorption (hsa04974), lysosomal activity (hsa04142), and apoptosis (hsa04210).

## Discussion

In line with the recommendations of stakeholders [5, 7], the objective of the current study was to identify a urinary proteomic biomarker that may predict 1-year post-ICU mortality, thereby revealing molecular pathways contributing to prognosis. Pursuing this objective, we identified the multidimensional urinary biomarker ACM128, consisting of 128 dysregulated peptide fragments. In multivariable-adjusted analyses, ACM128 measured at ICU admission predicted death within 1 year after ICU discharge with greater accuracy than clinical markers, such as the Charlson Comorbidity Index and the length of the ICU care, as well as other biomarkers tested within the context of post-ICU prognosis, including circulating BNP, hsTnT, active ADM and NGAL, and urinary albumin. Of note, the 1-year mortality was not significantly associated with circulating hsTnI, sST2, and urinary NGAL (Table 3).

A PubMed search with search terms “Intensive Care” AND “Mortality” AND “Proteome” OR “Proteomics” yielded 15 hits, published from 2003 until 2018, without any relevance to the current manuscript. Replacing “Proteome” by “Biomarker” yielded 420 hits, with few focusing on the biomarkers reported in Table 3 and none on a multidimensional proteomic marker. Previously published studies reported on NGAL [27], ADM [28, 29], NT-proB-type natriuretic peptide (NT-proBNP) [30, 31], hsTnT [32, 33], hsTnI [34], or sST2 used in combination with other circulating biomarkers [35]. In patients after major non-cardiac surgery, early NGAL-based prediction of imminent acute kidney injury followed by implementation of KDIGO care bundle (https://kdigo.org/guidelines/acute-kidney-injury/) reduced the severity of kidney injury, postoperative creatinine increase, and the length of ICU and hospital stay [27]. Compared with urinary NGAL, plasma NGAL was a better predictor of an adverse outcome (Table 3). Our observation replicated findings in 110 patients admitted to intensive care after cardiac surgery with cardiopulmonary bypass [36] and in 50 patients admitted to intensive care because of acute kidney injury [37]. Circulating NGAL is freely filtered in the glomeruli and reabsorbed in the proximal tubule [38, 39]. In response to kidney injury, NGAL expression is upregulated in particular in the distal tubules. However, NGAL passing the glomerular sieve and impaired proximal tubular reabsorption also contribute to the urinary NGAL level [40, 41]. On the other hand, NGAL back leaking from the tubules into the circulation, the extra-renal synthesis of NGAL, and reduced glomerular filtration all impact on the plasma levels [40, 41]. Thus, the kinetics of plasma and urinary NGAL explain why there might be discrepancy in the predictive value of both biomarkers.

In surgical patients with sepsis, ADM indicated a higher need of vasopressor treatment and predicted mortality after 90 days [28]. NT-proBNP is commonly elevated on admission to intensive care, increases with severity of disease, and is an independent predictor of mortality [42]. NT-proBNP and hsTnT exhibit additive prognostic potential, which exceeds their individual value. This might be attributed to a difference in underlying pathophysiological mechanisms and synergy between risk factors [30]. Furthermore, one study demonstrated that hsTNT was the only independent predictor of 1-year mortality in patients with shock, whereas BNP or echocardiographic indexes had no prognostic value [32, 33]. In patients with nontraumatic subarachnoid hemorrhage, hsTnI measured within 24 h after the event predicted the need for a higher inspiratory oxygen fraction [34]. HsTnI is also a sensitive, albeit unspecific marker of myocardial infarction. Our current findings move the field forward, because ACM128 was predictive of the 1-year mortality after ICU discharge, independent of the underlying condition requiring intensive care. Moreover, compared with the aforementioned biomarkers, ACM128 was a better prognosticator of death as evidenced by IDI, NRI, and ∆AUC. IDI and NRI thereby provided complementary information. Indeed, if addition of a biomarker to a model increases the predicted probability in cases, this is reflected by a significant increase in IDI. NRI indicates the extent by which a biomarker improves diagnostic accuracy [24].

Under physiological conditions, about 70% of the urinary proteome originates from the kidney and the urinary tract, while 30% is derived from plasma [43]. Approximately 60% of the total mass of urinary peptides and proteins consist of collagen fragments [44]. Interactome mapping, using parental proteins identified from sequenced peptides contained in ACM128 and the in silico predicted proteases, revealed as top deregulated pathways protein digestion and absorption, lysosomal activity, and apoptosis. Furthermore, there was limited overlap in the peptide fragments making up the ACM128 biomarker and those known to be associated with chronic kidney disease (the CKD273 panel) [45] or with coronary heart disease (the CAD238 panel) [46]. Thus, ACM128 does not merely reflect micro- or macro-vascular insult.

The analysis of sequenced peptides was collagen-driven, as reflected by predicted proteases and proteins assigned to enriched pathways. While the reduced number of peptides resulted, as expected, in a decrease in the pathway coverage, we observed high consistency in the interactome results irrespective of whether collagen fragments were included or not, supporting the validity of the approach.

Urinary proteomic profiling is progressing to clinical application for detection, prevention, and early intervention in type 2 diabetic patients with silent renal dysfunction [47, 48] and in patients at risk of left ventricular dysfunction [49, 50]. The multidimensional proteomic biomarker CKD273 (sensitivity/specificity > 90%) predicts the 3-year risk of kidney disease 2 years earlier than microalbuminuria (70%/45%) and leads to substantial cost savings [51], to be confirmed by the PRIORITY results to be published later this year [52, 53]. HF1 encompasses 85 urinary peptides and predicts subclinical diastolic left ventricular dysfunction 5 years ahead of echocardiography, while NT-proBNP or BNP are not diagnostic in asymptomatic patient at risk of heart failure [49, 50]. Our current findings highlight the need for further research into the clinical application of urinary proteomic profiling in ICU patients to identify those in need of further diagnostic and therapeutic work-up. Urinary proteomic profiling might replace magnetic resonance imaging [54] or kidney biopsy to diagnose myocardial and renal fibrosis, respectively. Furthermore, from the therapeutic angle, intensive risk factor management and antifibrotic drug treatment, using aldosterone receptor inhibitors, such as spironolactone or eplerenone, or the novel nonsteroidal anti-mineralocorticoid finerenone [55], or the novel sacubitril/valsartan [56], are options.

### Strong points and limitations

Strong points of the current study are its prospective design, the confirmation of the “a priori” hypothesis that multidimensional urinary biomarkers have clinical utility in risk stratification without the need to standardize for urinary volume or creatinine concentration. Patients randomly assigned to the discovery and test set (Additional file 1: Table S3) had similar characteristics. Our results replicate literature findings on atrial natriuretic peptides [30, 31], hsTnT [32, 33], active ADM [28, 29], and NGAL [27] and urinary albumin as predictors of outcome among ICU patients, but show that ACM128 outperforms all these markers in predicting 1-year mortality after ICU discharge. Notwithstanding these strengths, the current study must also be interpreted within the context of its limitations. First, we collected all-cause and not cause-specific mortality. However, all-cause mortality has the advantage that this outcome does not require any adjudication as cause-specific mortality does. Second, our study combines data from patients admitted to the ICU, because of surgical, medical, or mixed indications. While this may be considered as a weakness, it also facilitates the generalizability of our results. Finally, although the ACM128 outperformed established risk factors and widely used risk scores including Charlson Comorbidity Index and SOFA score as well as circulating and urinary biomarkers, cost-effectiveness analyses still have to be done as a lead on to further studies paving the way for the clinical application of ACM128.

## Conclusions and perspectives

The urinary proteomic classifier ACM128 predicts the 1-year post-ICU mortality over and beyond clinical risk factors and other biomarkers and revealed as top molecular pathways, potentially contributing to a fatal outcome, protein digestion and absorption, lysosomal activity, and apoptosis. The way to clinical application will be long and involves determining and validating diagnostic thresholds of ACM128, constructing risk prediction models, and last but not the least, if the previous steps are taken successfully, a randomized clinical trial following the PRIORITY design [52, 53].

## Supplementary information

**Additional file 1.** Supplemental Methods. Sample Preparation and CE-MS Analysis. Quality Control. Mass Spectrometric Data Processing. Support Vector Modelling. Sequencing of Polypeptides. References. **Table S1.** Weights of Comorbidities in the Charlson Comorbidity Index. **Table S2.** Correlation Coefficients between Biomarkers. **Table S3.** Clinical Characteristics at Baseline by Study Group and Survival Status. **Table S4.** Biomarkers at Baseline by Study Group and Survival Status. **Table S5.** Sequenced Peptides Included in the All-Cause Mortality Predictor Peptide Panel. **Table S6.** Association of Death with Single Sequenced Urinary Peptides. **Table S7.** Proteasix Analysis Including Collagen Fragments. Table S8 Proteasix Analysis Excluding Collagen Fragments. **Figure S1.** Distributions of multidimensional urinary ACM128 in survivors (A, C) and nonsurvivors (B, D) in the discovery and test datasets. **Figure S2.** Protein-protein interactome derived from 63 sequenced urinary peptides, including collagen fragments, and the in-silico Proteasix analysis. **Figure S3.** Protein-protein interactome derived from 35 sequenced urinary peptides, excluding collagen fragments, and the in-silico Proteasix analysis.

## Data Availability

All data will be available pending the consent from the patients.

## Abbreviations

ADM: Biologically active adrenomedullin
AUC: Area under the curve
BNP: Brain natriuretic peptide
CCI: Charlson Comorbidity Index
eGFRcrt: Estimated glomerular filtration rate was derived from serum creatinine
eGFRcys: Estimated glomerular filtration rate was derived from serum cystatin C
FROG-ICU: The French and European Outcome Registry in Intensive Care Unit
hsTnI: High-sensitive troponin I
hsTnT: High-sensitive troponin T
ICD: International Classification of Diseases
ICU: Intensive care units
IDI: Integrated discrimination improvement
NGAL: Neutrophil-gelatinase-associated lipocalin
NRI: Net reclassification improvement
NT proBNP: The N-terminal prohormone of brain natriuretic peptide
SOFA: The Sequential Organ Failure Assessment score
sST2: The interleukin 1 receptor family member soluble ST2
