# Supplementary material for: A novel urinary biomarker predicts 1-year mortality after discharge from intensive care
Source: Crit Care. 2020 Jan 9;24:10. doi: 10.1186/s13054-019-2686-0 (PMC6953276; doi:10.1186/s13054-019-2686-0)
Supplement: Supplementary file 1 — Additional file 1. Supplemental Methods. Sample Preparation and CE-MS Analysis. Quality Control. Mass Spectrometric Data Processing. Support Vector Modelling. Sequencing of Polypeptides. References. Table S1. Weights of Comorbidities in the Charlson Comorbidity Index. Table S2. Correlation Coefficients between Biomarkers. Table S3. Clinical Characteristics at Baseline by Study Group and Survival Status. Table S4. Biomarkers at Baseline by Study Group and Survival Status. Table S5. Sequenced Peptides Included in the All-Cause Mortality Predictor Peptide Panel. Table S6. Association of Death with Single Sequenced Urinary Peptides. Table S7. Proteasix Analysis Including Collagen Fragments. Table S8 Proteasix Analysis Excluding Collagen Fragments. Figure S1. Distributions of multidimensional urinary ACM128 in survivors (A, C) and nonsurvivors (B, D) in the discovery and test datasets. Figure S2. Protein-protein interactome derived from 63 sequenced urinary peptides, including collagen fragments, and the in-silico Proteasix analysis. Figure S3. Protein-protein interactome derived from 35 sequenced urinary peptides, excluding collagen fragments, and the in-silico Proteasix analysis. [file 13054_2019_2686_MOESM1_ESM.docx]

***CC***

**Additional file 1**

*A Novel Urinary Biomarker Predicts 1 Year Mortality after Discharge from Intensive Care*Esther Nkuipou-Kenfack, Agnieszka Latosinska, Wen-Yi Yang, Marie-Cécile Fournier, Elise Blet, Blerim Mujaj, Lutgarde Thijs, Elodie Feliot, Etienne Gayat, Harald Mischak, Jan A. Staessen, Alexandre Mebazaa,
Zhen-Yu Zhang, and the French and European Outcome Registry in Intensive Care Unit Investigators

**Table of Contents**

**Supplemental Methods**  p 2

Sample Preparation and CE-MS Analysis p 2

Quality Control p 3

Mass Spectrometric Data Processing p 3

Support Vector Modelling p 4

Sequencing of Polypeptides p 4

References p 5

**Table S1** Weights of Comorbidities in the Charlson Comorbidity Index p 8

**Table S2** Correlation Coefficients between Biomarkers p 9

**Table S3** Clinical Characteristics at Baseline by Study Group and Survival Status p10

**Table S4** Biomarkers at Baseline by Study Group and Survival Status p12

**Table S5** Sequenced Peptides Included in the All-Cause Mortality Predictor Peptide Panel p13

**Table S6** Association of Death with Single Sequenced Urinary Peptides p17

**Table S7** Proteasix Analysis Including Collagen Fragments p18

**Table S8** Proteasix Analysis Excluding Collagen Fragments p22

**Figure E1** Distributions of multidimensional urinary ACM128 in survivors (A, C) and
nonsurvivors (B, D) in the discovery and test datasets. p24

**Figure E2** Protein-protein interactome derived from 63 sequenced urinary peptides,
including collagen fragments, and the in-silico Proteasix analysis. p25

**Figure E3** Protein-protein interactome derived from 35 sequenced urinary peptides,
excluding collagen fragments, and the in-silico Proteasix analysis. p26

**Urinary Proteomics**

***Sample Preparation and CE-MS Analysis***

For proteomic analysis, a 0.7 mL aliquot of stored urine was thawed immediately before use and diluted with 0.7 mL of 2 M urea, 10 mM NH4OH containing 0.02% sodium dodecyl sulphate. To remove higher molecular mass proteins, such as albumin and immunoglobulins, the sample was ultra-filtered, using Centrisart ultracentrifugation filter devices (20 kDa MWCO; Sartorius, Göttingen, Germany) at 3000 relative centrifugal force units until 1.1 mL of filtrate was obtained. This filtrate was then applied onto a PD-10 desalting column (GE Healthcare, Uppsala, Sweden) equilibrated in 0.01% NH4OH in HPLC-grade in H2O (Carl Roth GmbH, Karlsruhe, Germany) to decrease matrix effects by removing urea, electrolytes, salts, and to enrich polypeptides. Finally, all samples were lyophilized, stored at 4°C, and suspended in HPLC-grade H2O shortly before CE-MS analyses.

Capillary electrophoresis coupled to mass spectrometry (CE-MS) was performed, using a P/ACE MDQ capillary electrophoresis system (Beckman Coulter, Fullerton, CA) on-line coupled to a micrOTOF MS (Bruker Daltonics, Bremen, Germany) (1. 2). The electrospray ionization device (Agilent Technologies, Palo Alto, CA) was grounded, and the ion spray interface potential was set between ‑4 and ‑4.5 kV. Data acquisition and MS acquisition methods were automatically controlled by the CE via contact-close-relays. Spectra were accumulated every 3 seconds over a mass-to-charge ratio (m/z) ranging from 350 to 3000.

***Quality Control***

Accuracy, precision, selectivity, sensitivity, reproducibility and stability of the CE-MS have been previously published (1. 3). Quality control involves daily CE-MS analysis of a human urine standard (3). To prevent variability due to carry-over effects from one to the next analysis, capillaries are reconditioned between runs with 1 M NaOH. The coefficient of variance estimated from over 600 human urine standard analyses for over 3 years was 5.8% (4).

***Mass Spectrometric Data Processing***

Mass spectral peaks representing identical molecules at different charge states were deconvoluted into single masses, using MosaiquesVisu software (5). Only signals with a charge > 1 observed in a minimum of three consecutive spectra with a signal-to-noise ratio of at least 4 were considered. Reference signals of 1770 urinary polypeptides were used for CE-time calibration by locally weighted regression. For normalization of analytical and urine dilution variances, signal intensities were normalized relative to 29 ‘‘*housekeeping*’’ peptides (6. 7). The obtained peak lists characterize each polypeptide by its molecular mass. normalized CE migration time and normalized signal intensity. All detected peptides were deposited, matched, and annotated in a Microsoft SQL database, allowing further statistical analysis (8). For clustering, peptides in different samples were considered identical, if mass deviation was less than 50 ppm. CE migration time was controlled to be below 0.35 minutes after calibration.

***Support Vector Modelling***

Support vector modelling (SVM) involves the classification of biomarker signals in samples in a high dimensional data space. The MosaCluster software, version 7.1.0, calculates classification scores based on the amplitudes of the biomarker (9. 10). The SVM classification score is generated by determining the Euclidian distance of the vector to a maximal margin hyperplane. The SVM classifier uses the logarithmically transformed intensities of peptides as coordinates in an x‑dimensional space. It then builds an x - 1 dimensional hyperplane by performing a quadratic programming optimization of a Lagrangian, using the training labels only, thereby allowing misclassification of samples in the hyperplane. To account for misclassification, SVM introduces a cost parameter C. Because non-separable problems in low dimensions may be separable in higher dimensions. SVM uses the Kernel-trick to transform samples to a higher dimensional space. MosaCluster uses the standard radial basis functions as kernel. These functions are just Gaussians with the parameter gamma controlling their width. The optimal parameters C and gamma were determined via a leave-one-out cross-validation error estimation.

***Sequencing of Polypeptides***

CE-MS signals were in silico assigned to the previously sequenced peptides from Human Urinary Proteome Database, version 2.0 (11). Peptides from this database were sequenced, as described elsewhere (12. 13). Briefly, urinary peptides were fragmented, using different tandem mass-spectrometric techniques with a prior separation step with CE or HPLC. Fragmentation spectra were matched to the protein sequences from up-to-date public databases (IPI, NCBI Reference Sequence Database and Uniprot), using MS/MS search engines MASCOT (Matrix Sciences Ltd., London, UK) and OMSSA (National Center for Biotechnology Information, Bethesda, MD). In matching, we accounted for urinary proteins post-translational modifications, such as hydroxylation of lysine and proline, and specific MS characteristics. Peptide sequences from LC-MS/MS analyses were verified by the comparison of experimental and theoretical CE migration time, which is dependent on the number of basic and neutral polar amino acids.

Identified specific urinary peptides were combined into multidimensional classifiers. using the support vector machine-based MosaCluster software, version 1.7.0 (14). MosaCluster calculates classification scores based on the amplitudes of the selected biomarkers. Classification is performed by determining the Euclidian distance (defined as the support-vector machine classification score) of the vector to a maximal margin hyperplane.

**References**

1. Theodorescu D. Wittke S. Ross MM. Walden M. Conaway M. Just I et al. Discovery and validation of new protein biomarkers for urothelial cancer : a prospective analysis. Lancet Oncol. 2006;7:230-40.

2. Wittke S. Mischak H. Walden M. Kolch W. Rädler T. Wiedemann K. Discovery of biomarkers in human urine and cerebrospinal fluid by capillary electrophoresis coupled to mass spectrometry: towards new diagnostic and therapeutic approaches. Electrophoresis. 2005;26:1476-87.

3. Mischak H. Kolch W. Aivalotis M. Bouyssie D. Court M. Dihazi H et al. Comprehensive human urine standards for comparability and standardization in clinical proteome analysis. Proteomics Clin Appl. 2010;4:464-78.

4. Mischak H. Vlahou A. Ioannidis JP. Technical aspects and inter-laboratory variability in native peptide profiling : the CE-MS experience. Clin Biochem. 2013;46:432-43.

5. Neuhoff NV. Kaiser T. Wittke S. Krebs R. Pitt A. Burchard A et al. Mass spectrometry for the detection of differentially expressed proteins : a comparison of surface-enhanced laser desorption/ionization and capillary electrophoresis/mass spectrometry. Rapid Commun Mass Spectrom. 2004;18:149-56.

6. Haubitz M. Good DM. Woywodt A. Haller H. Rupprecht H. Theodorescu D et al. Identification and validation of urinary biomarkers for differential diagnosis and evaluation of therapeutic intervention in anti-neutrophil cytoplasmic antibody-associated vasculitis. Moll Cell Proteomics. 2009;8:2296-307.

7. Jantos-Siwy J. Schiffer E. Brand K. Schumann G. Rossing K. Delles C et al. Quantitative urinary proteome analysis for biomarker evaluation in chronic kidney disease. J Proteome Res. 2009;8:268-81.

8. Dakna M. He Z. Yu WC. Mischak H. Kolch W. Technical. bioinformatical and statistical aspects of liquid chromatography-mass spectrometry (LC-MS) and capillary electrophoresis-mass spectrometry (CE-MS) based clinical proteomics: a critical assessment. J Chromatogr B Analyt Technol Biomed Life Sci. 2009;877:1250-8.

9. Girolami M. Mischak H. Krebs R. Analysis of complex multidimensional datasets. Technologies. 2006;3:13-9.

10. Mischak H. Allmaier G. Apweiler R. Attwood T. Baumann M. Benigni A et al. Recommendations for biomarker identification and qualification in clinical proteomics. Sci Transl Med. 2010;2:46ps42.

11. Stalmach A. Albalat A. Mullen W. Mischak H. Recent advances in capillary electrophoresis coupled to mass spectrometry for clinical proteomic applications. Electrophoresis. 2013;34:1452-64.

12. Coon JJ. Zürbig P. Dakna M. Dominiczak AF. Decramer S. Fliser D et al. CE-MS analysis of the human urinary proteome for biomarker discovery and disease diagnostics. Proteomics Clin Appl. 2008;2:964-73.

13. Rossing K. Mischak H. Dakna M. Zürbig P. Novak J. Julian BA et al. Urinary proteomics in diabetes and CKD. J Am Soc Nephrol. 2008;19:1283-90.

14. Delles C. Schiffer E. von Zur Muhlen C. Peter K. Rossing P. Parving HH et al. Urinary proteomic diagnosis of coronary artery disease: identification and clinical validation in 623 individuals. J Hypertens. 2010;28:2316-22.

**Table S1**

**.** Weights of Comorbidities in the Charlson Comorbidity Index

| Comorbidities | Charlson Comorbidity Index weight |
| --- | --- |
| Myocardial infarction | 1 |
| Congestive heart failure | 1 |
| Peripheral vascular disease | 1 |
| Cerebrovascular disease | 1 |
| Dementia | 1 |
| Chronic obstructive pulmonary disease | 1 |
| Connective tissue disease | 1 |
| Peptic ulcer disease | 1 |
| Mild liver disease | 1 |
| Diabetes mellitus without end‐organ damage | 1 |
| Hemiplegia | 2 |
| Moderate to severe chronic kidney disease | 2 |
| Diabetes with end‐organ damage | 2 |
| Solid tumor | 2 |
| Leukemia | 2 |
| Lymphoma | 2 |
| Moderate to severe liver disease | 3 |
| Metastatic solid tumor | 6 |
| Acquired immunodeficiency syndrome | 6 |

Reproduced from J Chronic Dis. 1987;40(5):373-83.

**Table S2**

**.** Correlation Coefficients between Biomarkers

| Biomarkers | | Circulating | | | | | | Urinary | | |
| --- | --- | --- | --- | --- | --- | --- | --- | --- | --- | --- |
|  |  | BNP | hsTnT | hsTnI | ADM | sST2 | NGAL | Albuminuria | NGAL | ACM128 |
| Circulating | BNP | … | 0.55‡ | 0.51‡ | 0.42‡ | 0.36‡ | 0.40‡ | 0.04 | 0.36‡ | 0.42‡ |
|  | hsTnT | 0.55‡ | … | 0.85‡ | 0.34‡ | 0.27‡ | 0.30‡ | -0.07* | 0.26‡ | 0.31‡ |
|  | hsTnI | 0.51‡ | 0.85‡ | … | 0.24‡ | 0.29‡ | 0.21‡ | -0.07* | 0.19‡ | 0.19‡ |
|  | ADM | 0.41‡ | 0.34‡ | 0.24‡ | … | 0.45‡ | 0.59‡ | -0.008 | 0.57‡ | 0.43‡ |
|  | sST2 | 0.36‡ | 0.27‡ | 0.29‡ | 0.45‡ | … | 0.49‡ | 0.11‡ | 0.45‡ | 0.19‡ |
|  | NGAL | 0.40‡ | 0.30‡ | 0.21‡ | 0.59‡ | 0.49‡ | … | 0.11‡ | 0.62‡ | 0.45‡ |
| Urinary | Albuminuria | 0.04 | -0.07* | ‑0.07* | ‑0.008 | 0.11‡ | 0.11‡ | … | 0.015 | 0.038 |
|  | NGAL | 0.36‡ | 0.26‡ | 0.19‡ | 0.57‡ | 0.45‡ | 0.62‡ | 0.015 | … | 0.42‡ |
|  | ACM128 | 0.42‡ | 0.31‡ | 0.19‡ | 0.43‡ | 0.19‡ | 0.45‡ | 0.038 | 0.42‡ | … |

Abbreviations and units of the biomarkers are given in Table 2.
Significance of the correlation coefficients: * *P* ≤ 0·05; † *P* ≤ 0·01; ‡ *P* *≤* 0·0001

**Table S3**

**.** Clinical Characteristics at Baseline by Study Group and Survival Status (Starts)

| Characteristic |  | Discovery Phase | | |  | Test Phase | | |
| --- | --- | --- | --- | --- | --- | --- | --- | --- |
|  |  | Survivors  (*n* = 299) | Nonsurvivors  (*n* = 70) | *P* Value |  | Survivors  (*n* = 699) | Nonsurvivors  (*n* = 175) | *P* Value |
| N° with characteristic (%) |  |  |  |  |  |  |  |  |
| Women |  | 110 (36.8) | 24 (34.3) | 0.70 |  | 247 (35.3) | 62 (35.4) | 0.98 |
| Diabetes mellitus |  | 44 (14.7) | 24 (34.3) | 0.0001 |  | 101 (14.5) | 41 (23.4) | 0.004 |
| Indication of intensive care |  |  |  |  |  |  |  |  |
| Acute respiratory insufficiency |  | 56 (18.7) | 17 (24.3) | 0.29 |  | 142 (20.3) | 46 (26.3) | 0.086 |
| Pancreatitis or liver failure |  | 2 (0.7) | 3 (4.3) | 0.019 |  | 12 (1.7) | 5 (2.9) | 0.33 |
| Hemorrhagic or hypovolemic shock |  | 17 (5.7) | 3 (4.3) | 0.64 |  | 39 (5.6) | 18 (10.3) | 0.024 |
| Cardiogenic shock or heart failure |  | 44 (14.7) | 10 (14.3) | 0.93 |  | 101 (14.5) | 24 (13.7) | 0.80 |
| Sepsis or anaphylactic shock |  | 73 (24.4) | 22 (31.4) | 0.23 |  | 157 (22.5) | 46 (26.3) | 0.28 |
| Post-surgical care |  | 41 (13.7) | 5 (7.1) | 0.13 |  | 60 (8.6) | 18 (10.3) | 0.48 |
| Severe trauma |  | 20 (6.7) | 0 | 0.026 |  | 58 (8.3) | 0 | <0.0001 |
| Other indications |  | 46 (15.4) | 10 (14.3) | 0.82 |  | 130 (18.6) | 18 (10.3) | 0.0087 |
| Treatment administered |  |  |  |  |  |  |  |  |
| Mechanical ventilation |  | 189 (63.2) | 39 (55.7) | 0.25 |  | 426 (60.9) | 100 (57.1) | 0.36 |
| Extracorporeal membrane oxygenation |  | 3 (1.0) | 0 | 0.40 |  | 6 (0.9) | 1 (0.6) | 0.70 |
| Dialysis |  | 16 (5.4) | 8 (11.4) | 0.063 |  | 41 (5.9) | 17 (9.7) | 0.067 |

**Table S3**

**.** Clinical Characteristics at Baseline by Study Group and Survival Status (Continued)

| Characteristic |  | Discovery Phase | | |  | Test Phase | | |
| --- | --- | --- | --- | --- | --- | --- | --- | --- |
|  |  | Survivors  (*n* = 256) | Nonsurvivors  (*n* = 66) | *P* |  | Survivors  (*n* = 608) | Nonsurvivors  (*n* = 151) | *P* |
| Mean characteristic (±SD) |  |  |  |  |  |  |  |  |
| Age, y |  | 57.0 (17.1) | 70.3 (13.0) | <0.0001 |  | 57.3 (16.9) | 68.4 (11.9) | <0.0001 |
| Body mass index, kg/m2 |  | 27.2 (7.1) | 24.6 (3.8) | 0.031 |  | 27.9 (7.8) | 27.4 (5.7) | 0.58 |
| Systolic pressure, mm Hg |  | 125.9 (23.0) | 121.9 (21.2) | 0.19 |  | 125.6 (22.3) | 125.6 (23.1) | 0.89 |
| Diastolic pressure, mm Hg |  | 64.6 (13.4) | 58.3 (11.9) | 0.0004 |  | 64.4 (14.2) | 63.0 (13.4) | 0.23 |
| Mean arterial pressure, mm Hg |  | 85.0 (14.5) | 79.5 (12.3) | 0.0037 |  | 84.8 (14.8) | 83.9 (14.0) | 0.44 |
| Heart rate, beats per minute |  | 92.4 (20.5) | 87.4 (20.2) | 0.069 |  | 92.6 (20.8) | 90.2 (19.5) | 0.17 |
| Blood glucose, mmol/L |  | 7.18 (2.61) | 7.22 (2.16) | 0.19 |  | 7.42 (2.20) | 7.84 (2.89) | 0.15 |
| eGFRcrt, mL/min/1.73 m2 |  | 95.7 (55.5) | 73.3 (49.7) | 0.0021 |  | 97.3 (55.7) | 82.9 (60.8) | 0.0027 |
| eGFRcys, mL/min/1.73 m2 |  | 77.2 (49.3) | 51.8 (43.4) | <0.0001 |  | 75.2 (44.4) | 47.0 (32.9) | <0.0001 |
| Median characteristic (IQR) |  |  |  |  |  |  |  |  |
| Charlson score |  | 2 (0–4) | 5 (3–6) | <0.0001 |  | 2 (1–4) | 4 (3–6) | <0.0001 |
| SOFA score |  | 7 (5–10) | 7 (4–9) | 0.11 |  | 7 (4–10) | 8 (5–10) | 0.27 |
| Length of ICU stay, days |  | 12 (7–17) | 10 (6–21) | 0.071 |  | 11 (6–18) | 13 (7–23) | 0.036 |

Abbreviations: eGFRcrt/eGFRcys, estimated glomerular filtration rate derived from serum creatinine/cystatin C according to the Chronic Kidney Disease Epidemiology Collaboration equation (16); ICU, intensive care unit; SOFA, Sequential Organ Failure Assessment Score (15); IQR, interquartile range; . Baseline refers to the date of discharge from the Intensive care unit. Body mass index was body weight in kilograms divided by height in meters squared. Mean arterial pressure in diastolic pressure plus one third of the difference between systolic and diastolic pressure. Diabetes mellitus was a fasting or random glucose ≥ 7.0/11.1 mmol/L, use of antidiabetic agents or a diagnosis in medical records. *P* denotes the significance of the difference between survivors and nonsurvivors.

**Table S4**

**.** Biomarkers at Baseline by Study Group and Survival Status

| Biomarkers |  | Discovery Phase | | |  | Test Phase | | |
| --- | --- | --- | --- | --- | --- | --- | --- | --- |
|  |  | Survivors  (*n* = 299) | Nonsurvivors  (*n* = 70) | *P* |  | Survivors  (*n* = 699) | Nonsurvivors  (*n* = 151) | *P* |
| Circulating |  |  |  |  |  |  |  |  |
| BNP, pg/L |  | 109 (32, 302) | 300 (100, 815) | <0.0001 |  | 105 (35, 303) | 233 (86, 669) | <0.0001 |
| hsTNT, pg/L |  | 34 (13, 112) | 43 (19, 296) | 0.029 |  | 31 (12, 82) | 47 (19, 171) | <0.0001 |
| hsTnI, pg/L |  | 33 (7, 201) | 39 (14, 485) | 0.023 |  | 27 (8, 177) | 42 (13, 390) | 0.0078 |
| ADM, nmol/L |  | 47 (29, 85) | 85 (42, 166) | 0.0002 |  | 51 (29, 94) | 78 (42, 142) | <0.0001 |
| sST2, ng/mL |  | 288 (179, 523) | 408 (236, 981) | 0.0086 |  | 280 (155, 489) | 343 (188, 844) | 0.0058 |
| NGA L, mg/mL |  | 157 (83, 309) | 297 (145, 599) | <0.0001 |  | 145 (80, 303) | 256 (121, 510) | <0.0001 |
| Urinary |  |  |  |  |  |  |  |  |
| Albuminuria, mg/L |  | 384 (298, 467) | 407 (348, 478) | 0.12 |  | 381 (300, 467) | 380 (284, 455) | 0.60 |
| NGAL ng/mL |  | 97 (31, 460) | 221 (65, 1374) | 0.0005 |  | 81 (31, 360) | 196 (42, 654) | 0.0003 |
| ACM128 |  | ‑0.59 (‑1.00, ‑0.15) | 0.63 (‑0.01, 1.00) | <0.0001 |  | ‑0.49 (‑0.85, ‑0.02) | 0.01 (‑0.43, 0.41) | <0.0001 |

Abbreviations: NT‑proBNP, N‑terminal pro-atrial natriuretic peptide; hsTnT, high-sensitive troponin T; hsTnI, high-sensitive troponin I; ADM, biologically active adrenomedullin [ADM]; sST2, soluble ST2; NGAL, neutrophil-gelatinase-associated lipocalin; ACM128, multidimensional urinary proteomic biomarker. Values are medians (interquartile range). *P*denotes the significance of the difference between survivors and nonsurvivors.

**Table S5**

**.** Sequenced Peptides Included in the All-Cause Mortality Predictor Peptide Panel (Starts)

| ID | Sequence | Protein name | Accession number | Controls | | Cases | | R |
| --- | --- | --- | --- | --- | --- | --- | --- | --- |
|  |  |  |  | % | MA | % | MA |  |
| e19541 | EVGKpGERGLHGEFGLPGPAGpRGERGpPGESGAAGPTGPIG | Collagen alpha-2(I) chain | P08123 | 35.0 | 87.0 | 18.8 | 30.7 | 0.35 |
| e04647 | DGPpGRDGQpGHKG | Collagen alpha-2(I) chain | P08123 | 35.1 | 29.1 | 18.4 | 12.8 | 0.44 |
| e10070 | DEAGSEADHEGTHSTKRGH | Fibrinogen alpha chain | P02671 | 33.7 | 45.8 | 20.0 | 21.7 | 0.47 |
| e03055 | GEAGHPGPPGPpGP | Collagen alpha-1(V) chain | P20908 | 53.5 | 269.7 | 37.1 | 142.7 | 0.53 |
| e17825 | GPpGADGQPGAKGEPGDAGAKGDAGPPGpAGPAGPpGPIG | Collagen alpha-1(I) chain | P02452 | 51.6 | 259.8 | 39.2 | 149.5 | 0.58 |
| e12154 | GEWKEGEKDPWGVSMMNTSF | MORN repeat-containing protein 4 | Q8WVZ3 | 35.3 | 185.8 | 26.5 | 113.2 | 0.61 |
| e13825 | LTGPIGppGPAGAPGDKGESGPSGPAGPTG | Collagen alpha-1(I) chain | P02452 | 80.5 | 701.8 | 69.0 | 469.1 | 0.67 |
| e07461 | TLSQPKIVKWDRDM | Beta-2-microglobulin | P61769 | 16.8 | 372.6 | 24.5 | 249.6 | 0.67 |
| e11415 | IGPpGPAGApGDKGESGPSGPAGPTG | Collagen alpha-1(I) chain | P02452 | 71.0 | 338.4 | 58.0 | 229.8 | 0.68 |
| e15411 | PQGPpGPTGpGGDKGDTGPpGPQGLQGLpGT | Collagen alpha-1(III) chain | P02461 | 56.3 | 342.9 | 44.9 | 240.3 | 0.70 |
| e16933 | GpQGFQGPAGEPGEPGQTGPAGARGpAGpPGKAGE | Collagen alpha-2(I) chain | P08123 | 29.7 | 30.2 | 20.8 | 22.5 | 0.74 |
| e17946 | PpGPAGFAGPPGADGQPGAKGEpGDAGAKGDAGPPGPAGP | Collagen alpha-1(I) chain | P02452 | 85.6 | 920.8 | 76.7 | 695.8 | 0.76 |
| e06256 | TGLSMDGGGSPKGDVDP | Na/K-transporting ATPase subunit gamma | P54710 | 38.2 | 107.5 | 29.0 | 82.6 | 0.77 |
| e05423 | VGpPGPPGpPGPPGPPS | Collagen alpha-1(I) chain | P02452 | 63.0 | 445.0 | 53.5 | 347.5 | 0.78 |
| e08463 | DEAGSEADHEGTHSTKR | Fibrinogen alpha chain | P02671 | 88.6 | 2095.6 | 82.4 | 1662.2 | 0.79 |
| e07093 | VIDQSRVLNLGPITR | Uromodulin | P07911 | 70.4 | 5308.0 | 66.5 | 4476.4 | 0.84 |
| e19228 | PGLPGPSGEpGKQGpSGASGERGPPGPMGPPGLAGppGESGR | Collagen alpha-1(I) chain | P02452 | 52.4 | 117.5 | 45.3 | 100.2 | 0.85 |
| e11213 | ADGQPGAKGEpGDAGAKGDAGPPGP | Collagen alpha-1(I) chain | P02452 | 67.5 | 444.2 | 55.5 | 404.1 | 0.91 |
| e09874 | DGESGRpGRPGERGLpGPpG | Collagen alpha-1(III) chain | P02461 | 92.4 | 2402.7 | 93.1 | 2581.8 | 1.07 |

**Table S5.** Sequenced Peptides Included in the All-Cause Mortality Predictor Peptide Panel (Continued)

| ID | Sequence | Protein name | Accession number | Controls | | Cases | | R |
| --- | --- | --- | --- | --- | --- | --- | --- | --- |
|  |  |  |  | % | MA | % | MA |  |
| e00152 | VSGFHPSD | Beta-2-microglobulin | P61769 | 32.8 | 192.3 | 31.4 | 217.7 | 1.13 |
| e09989 | DGESGRpGRpGERGLpGPpG | Collagen alpha-1(III) chain | P02461 | 95.3 | 5151.5 | 93.5 | 5856.9 | 1.14 |
| e04531 | ATSFRR | Protein shroom3 | Q8TF72 | 86.9 | 1743.8 | 84.1 | 2042.4 | 1.17 |
| e06708 | GAkGDAGpAGpkGEPGSP | Collagen alpha-1(I) chain | P02452 | 96.5 | 6089.5 | 95.9 | 7465.8 | 1.23 |
| e06457 | NGERIEKVEHSDLS | Beta-2-microglobulin | P61769 | 26.6 | 206.4 | 27.8 | 258.3 | 1.25 |
| e11661 | GERIEKVEHSDLSFSKDWS | Beta-2-microglobulin | P61769 | 24.6 | 57.1 | 29.0 | 72.5 | 1.27 |
| e07972 | GEKGpSGEAGTAGPpGTpGP | Collagen alpha-2(I) chain | P08123 | 27.1 | 180.7 | 31.0 | 235.1 | 1.30 |
| e14398 | PGMPGADGpPGHPGKEGppGEKGGQGpPG | Collagen alpha-1(V) chain | P20908 | 92.8 | 1599.1 | 94.7 | 2126.0 | 1.33 |
| e06355 | DpGPpGQSGRDGYPGp | Collagen alpha-1(XXIII) chain | Q86Y22 | 53.1 | 113.0 | 51.4 | 158.0 | 1.40 |
| e07132 | EpGSpGENGAPGQmGPR | Collagen alpha-1(I) chain | P02452 | 33.7 | 782.9 | 38.8 | 1144.3 | 1.46 |
| e05269 | DGLAHLDNLKGTFA | Hemoglobin subunit beta | P68871 | 19.1 | 68.1 | 22.9 | 102.7 | 1.51 |
| e11518 | GDAGNSIGGGRGEpGppGLpGPPGP | Collagen alpha-1(XIII) chain | Q5TAT6 | 23.0 | 34.9 | 26.1 | 52.7 | 1.51 |
| e11611 | IRGPQGHQGPAGPpGPpGPpGPpG | Collagen alpha-2(I) chain | P08123 | 42.2 | 226.8 | 52.6 | 345.0 | 1.52 |
| e12939 | LQGTPVAQMTEDAVDAERLKHL | Complement C3 | P01024 | 37.1 | 608.0 | 42.0 | 934.7 | 1.54 |
| e12518 | DAHKSEVAHRFKDLGEENFK | Serum albumin | P02768 | 48.7 | 2432.9 | 57.5 | 3747.8 | 1.54 |
| e14660 | RLTWASHEKMHEGDEGPGHHHKPG | Protein S100-A9 | P06702 | 33.1 | 150.2 | 35.1 | 232.7 | 1.55 |
| e14159 | SNGADLSGVTEEAPLKLSKAVHKAVL | Alpha-1-antitrypsin | P01009 | 20.4 | 186.4 | 29.4 | 293.9 | 1.58 |
| e09616 | ASAGTGDLSDNHDIISmKL | Vesicular integral-membrane protein VIP36 | Q12907 | 24.5 | 177.4 | 29.4 | 281.2 | 1.59 |
| e11562 | LENEDRRSASLHLPKLSITG | Alpha-1-antitrypsin | P01009 | 27.2 | 475.8 | 37.1 | 756.8 | 1.59 |
| e11768 | pGFPGAQGEPGSQGEpGDpGLpGP | Collagen alpha-2(IV) chain | P08572 | 28.6 | 101.6 | 31.8 | 165.2 | 1.63 |

**Table S5.** Sequenced Peptides Included in the All-Cause Mortality Predictor Peptide Panel (Continued)

| ID | Sequence | Protein name | Accession number | Controls | | Cases | | R |
| --- | --- | --- | --- | --- | --- | --- | --- | --- |
|  |  |  |  | % | MA | % | MA |  |
| e11904 | GPAGFpGApGQNGEpGGKGERGApG | Collagen alpha-1(III) chain | P02461 | 46.6 | 137.3 | 55.1 | 223.7 | 1.63 |
| e05683 | SALEEYTKKLNTQ | Apolipoprotein A | F8W696 | 54.0 | 509.9 | 58.8 | 833.2 | 1.63 |
| e09697 | PGpPGPHGPPGpmGPHGLpGP | Collagen alpha-1(XXV) chain | Q9BXS0 | 53.9 | 223.8 | 55.9 | 382.8 | 1.71 |
| e14697 | FLPDEGKLQHLENELTHDIITKF | Alpha-1-antitrypsin | P01009 | 37.9 | 3403.2 | 47.8 | 5832.2 | 1.71 |
| e13865 | LPDEGKLQHLENELTHDIITKF | Alpha-1-antitrypsin | P01009 | 40.5 | 1349.5 | 46.1 | 2373.9 | 1.76 |
| e09750 | SGPVGppGLAGERGEQGppGP | Collagen alpha-2(V) chain | P05997 | 19.7 | 51.2 | 29.0 | 91.1 | 1.78 |
| e17164 | TVNFGDTEEAKKQINDYVEKGTQGKIVDL | Alpha-1-antitrypsin | P01009 | 22.7 | 862.2 | 30.2 | 1612.2 | 1.87 |
| e20740 | SDKPDMAEIEKFDKSKLKKTETQEKNPLPSKETIEQEKQAGES | Thymosin beta-4 | P62328 | 67.1 | 5737.7 | 67.8 | 11152.3 | 1.94 |
| e00274 | TVEAPMPK | Heat shock protein beta-1 | P04792 | 30.8 | 101.2 | 32.2 | 196.7 | 1.94 |
| e08700 | SGGSRSFSTASAITPSVSR | Keratin; type II cytoskeletal 5 | F8VU69 | 24.6 | 168.0 | 29.0 | 327.3 | 1.95 |
| e10277 | nGDDGEAGKpGRPGERGPPGp | Collagen alpha-1(I) chain | P02452 | 83.1 | 1407.7 | 88.2 | 2776.5 | 1.97 |
| e00883 | VIVKPHDPA | Cornulin | Q9UBG3 | 31.2 | 449.5 | 38.0 | 901.2 | 2.00 |
| e05509 | SQPKIVKWDRDM | Beta-2-microglobulin | P61769 | 43.8 | 432.41 | 52.2 | 871.7 | 2.02 |
| e19300 | NHANHTGSNHTYLKNTYNKPKLSEPEEELLQQF | Inner nuclear membrane protein Man1 | Q9Y2U8 | 50.9 | 1222.6 | 53.5 | 2497.2 | 2.04 |
| e04988 | TIDEKGTEAAGAMF | Alpha-1-antitrypsin | P01009 | 26.0 | 566.5 | 32.6 | 1179.6 | 2.08 |
| e20748 | EIGAKGSKGYQGNSGAPGSPGVKGAKGGpGpRGpKGEPGRRGDPGTKGSpGSD | Collagen alpha-2(VI) chain | P12110 | 41.2 | 1051.3 | 43.7 | 2211.8 | 2.10 |
| e12970 | DAHKSEVAHRFKDLGEENFKA | Serum albumin | P02768 | 47.4 | 1181.9 | 55.5 | 2525.3 | 2.14 |
| e11679 | EGpPGKPGEDGEpGRNGNpGEVG | Collagen alpha-2(V) chain | P05997 | 57.0 | 762.4 | 60.0 | 1688.1 | 2.21 |

**Table S5**

**.** Sequenced Peptides Included in the All-Cause Mortality Predictor Peptide Panel (Ends)

| ID | Sequence | Protein name | Accession number | Controls | | Cases | | R |
| --- | --- | --- | --- | --- | --- | --- | --- | --- |
|  |  |  |  | % | MA | % | MA |  |
| e11969 | KGPDGTPGEDGGEPGDAVAAAEQPA | F-box only protein 31 | Q5XUX0 | 20.5 | 25.8 | 26.9 | 57.9 | 2.25 |
| e11723 | IEQNTKSPLFMGKVVNPTQK | Alpha-1-antitrypsin | P01009 | 29.5 | 910.7 | 35.5 | 2057.4 | 2.26 |
| e18378 | EDPQGDAAQKTDTSHHDQDHPTFNKITPNLAE | Alpha-1-antitrypsin | P01009 | 56.1 | 3075.6 | 60.4 | 7095.3 | 2.31 |
| e01274 | RVAPEEHPV | POTE ankyrin domain family member F | A5A3E0 | 75.0 | 1443.9 | 76.7 | 3573.6 | 2.48 |
| e13226 | PDEGKLQHLENELTHDIITKF | Alpha-1-antitrypsin | P01009 | 24.1 | 261.2 | 36.1 | 647.7 | 2.48 |
| e12911 | FKGKWERPFEVKDTEEEDF | Alpha-1-antitrypsin | P01009 | 54.0 | 294.6 | 60.0 | 768.3 | 2.61 |

The accession number is the identifier in the UniProtKB database ([www.uniprot.org](http://www.uniprot.org)). Amplitude refers to the average mass spectrometric signal and frequency to the number of patients with a detectable signal. MA. mean signal amplitude of the polypeptides. R was calculated as mean amplitude in 245 nonsurvivors (cases) divided by mean amplitude in 998 survivors (controls). The polypeptides were ordered by ascending R.

**Table S6**

**.** Association of Death with Single Sequenced Urinary Peptides

| ID | Parental protein |  | HR (95% CI) | *P* |
| --- | --- | --- | --- | --- |
| e10277 | Collagen alpha-1 (I) chain |  | 1.50 (1.31–1.68) | <0.0001 |
| e10953 | Collagen alpha-1 (I) chain |  | 1.30 (1.11–1.48) | <0.0001 |
| e11452 | Collagen alpha-1 (I) chain |  | 1.28 (1.10–1.46) | <0.0001 |
| e14398 | Collagen alpha-1 (V) chain |  | 1.29 (1.10–1.47) | 0.00014 |
| e15237 | Collagen alpha-1 (III) chain |  | 1.28 (1.10–1.46) | 0.00017 |
| e15863 | Collagen alpha-2 (I) chain |  | 1.26 (1.08–1.43) | 0.00031 |
| e10863 | Collagen alpha-1 (I) chain |  | 1.25 (1.07–1.43) | 0.00059 |
| e11325 | Collagen alpha-1 (I) chain |  | 1.23 (1.06–1.41) | 0.00093 |
| e11013 | Collagen alpha-1 (I) chain |  | 1.24 (1.06–1.42) | 0.0010 |
| e11668 | Collagen alpha-1 (III) chain |  | 1.23 (1.05–1.42) | 0.0011 |
| e08463 | Fibrinogen alpha chain |  | 0.80 (0.60–0.99) | 0.0013 |
| e10025 | Collagen alpha-1 (I) chain |  | 1.22 (1.04–1.40) | 0.0019 |
| e10266 | Collagen alpha-1 (I) chain |  | 1.21 (1.03–1.38) | 0.0023 |
| e11122 | Collagen alpha-1 (I) chain |  | 1.22 (1.03–1.40) | 0.0026 |
| e10395 | Collagen alpha-1 (I) chain |  | 1.21 (1.03–1.39) | 0.0030 |
| e16118 | Collagen alpha-1 (I) chain |  | 1.20 (1.03–1.38) | 0.0034 |
| e10554 | Collagen alpha-1 (I) chain |  | 1.21 (1.03–1.39) | 0.0035 |
| e10502 | Collagen alpha-1 (III) chain |  | 1.19 (1.02–1.37) | 0.0049 |
| e11375 | Collagen alpha-1 (I) chain |  | 1.20 (1.02–1.38) | 0.0050 |

Analysis included 153 sequenced peptides with detectable signal in 70% of patients. Hazard ratios (HR), given with 95% confidence interval (CI) express the risk for a 1‑SD increment in the peptide signal amplitude. HRs were adjusted for center, sex, age, mean arterial pressure, eGFRcys and diabetes mellitus. Confidence intervals and *P* values were corrected for multiple testing using the Bonferroni approach, using the number of parental proteins identified (n = 10).

**Table S7**

**.** Proteasix Analysis Including Collagen Fragments (Starts)

| Protease (Gene) | N° of Cleaving Sites | | Cleaved Proteins | Δ | *P* |
| --- | --- | --- | --- | --- | --- |
|  | ↓ | ↑ |  |  |  |
| Cathepsin E (CTSE) | 1 | 22 | SERPINA1 (13), C3 (2), HSPB1 (1), CRNN (1), POTEF (1), APOA1 (1), LMAN2 (1), COL5A2 (1), ALB (1), MORN4 (1) | 1.98 | <0.0001 |
| Cathepsin D (CTSD) | 3 | 23 | SERPINA1 (13), C3 (2), HSPB1 (1), POTEF (1), COL5A2 (1), ALB (1), LEMD3 (1), CRNN (1), APOA1 (1), LMAN2 (1), COL1A1 (1), MORN4 (1), COL1A2 (1) | 1.89 | <0.0001 |
| Kallikrein-6 (KLK6) | 2 | 9 | COL1A1 (2), COL1A2 (2), HSPB1 (1), APOA1 (1), KRT5 (1), COL5A2 (1), COL13A1 (1), COL3A1 (1), SERPINA1 (1) | 1.77 | <0.0001 |
| Cathepsin G (CTSG) | 2 | 23 | SERPINA1 (11), POTEF (2), COL1A1 (2), LEMD3 (1), B2M (1), CRNN (1), KRT5 (1), HSPB1 (1), APOA1 (1), COL1A2 (1), SHRM3 (1), MORN4 (1), COL5A1 (1) | 1.7 | <0.0001 |
| Neutrophil elastase (ELANE) | 6 | 19 | COL1A1 (5), SERPINA1 (4), HBB (2), B2M (2), FBXO31 (2), HSPB1 (1), CRNN (1), POTEF (1), APOA1 (1), COL1A2 (1), KRT5 (1), COL5A2 (1), COL13A1 (1), COL3A1 (1), LEMD3 (1) | 1.67 | <0.0001 |
| Cathepsin S (CTSS) | 20 | 42 | COL1A1 (13), SERPINA1 (10), COL1A2 (8), COL3A1 (4), B2M (3), HBB (2), FGA (2), KRT5 (2), FBXO31 (2), MORN4 (2), COL6A2 (2), HSPB1 (1), CRNN (1), POTEF (1), COL5A1 (1), SHRM3 (1), APOA1 (1), LMAN2 (1), COL13A1 (1), COL5A2 (1), ALB (1), C3 (1), S100A9 (1) | 1.52 | <0.0001 |
| Cathepsin L1 (CTSL) | 16 | 41 | COL1A1 (12), COL1A1 (10), SERPINA1 (7), COL1A2 (5), COL3A1 (4), B2M (2), COL5A1 (2), HBB (2), COL6A2 (1), HSPB1 (1), CRNN (1), SHRM3 (1), APOA1 (1), KRT5 (1), LMAN2 (1), COL13A1 (1), COL5A2 (1), FBXO31 (1), MORN4 (1), ALB (1), C3 (1) | 1.51 | <0.0001 |

↓ ↑ Δ indicate downregulated and upregulated cleaving sites and the nonsurvivor/survivor ratio, respectively. Bracketed numbers indicate the number of cleaved proteins.

**Table S7**

**.** Proteasix Analysis Including Collagen Fragments (Continued)

| Protease (Gene) | N° of Cleaving Sites | | Cleaved Proteins | Δ | *P* |
| --- | --- | --- | --- | --- | --- |
|  | ↓ | ↑ |  |  |  |
| Neutrophil collagenase (MMP8) | 7 | 18 | COL1A1 (7), COL1A2 (4), SERPINA1 (3), COL3A1 (3), B2M (1), HSPB1 (1), COL25A1 (1), COL5A2 (1), ALB (1), S100A9 (1), LEMD3 (1), COL6A2 (1) | 1.44 | <0.0001 |
| Cathepsin K (CTSK) | 13 | 33 | COL1A1 (9), SERPINA1 (6), COL1A2 (5), COL3A1 (3), B2M (2), COL6A2 (2), HSPB1 (2), HBB (2), FGA (2), POTEF (1), FBXO31 (1), COL5A2 (1), CRNN (1), KRT5 (1), APOA1 (1), LMAN2 (1), S100A9 (1), ALB (1), SHRM3 (1), UMOD (1), MORN4 (1), COL5A1 (1) | 1.43 | <0.0001 |
| 72 kDa type IV collagenase (MMP2) | 7 | 21 | COL1A2 (8), COL1A1 (5), COL3A1 (3), SERPINA1 (2), B2M (1), CRNN (1), POTEF (1), SHRM3 (1), COL23A1 (1), UMOD (1), COL25A1 (1), COL5A2 (1), ALB (1), COL6A2 (1) | 1.43 | <0.0001 |
| Cathepsin B (CTSB) | 15 | 31 | COL1A1 (11), COL3A1 (6), COL1A2 (6), SERPINA1 (4), COL6A2 (2), KRT5 (2), HBB (2), HSPB1 (1), COL5A2 (1), ALB (1), CRNN (1), APOA1 (1), LMAN2 (1), S100A9 (1), C3 (1), COL13A1 (1), COL23A1 (1), COL5A1 (1), SHRM3 (1), MORN4 (1) | 1.42 | <0.0001 |
| Meprin A subunit alpha (MEP1A) | 21 | 45 | COL1A1 (12), SERPINA1 (11), COL1A2 (8), COL3A1 (6), B2M (3), FGA (3), CRNN (2), HBB (2), KRT5 (2), COL5A2 (2), ALB (2), C3 (2), COL6A2 (2), HSPB1 (1), COL5A1 (1), SHRM3 (1), APOA1 (1), COL23A1 (1), UMOD (1), FBXO31 (1), MORN4 (1), S100A9 (1) | 1.41 | <0.0001 |
| A disintegrin and metalloproteinase with thrombospondin motifs 4 (ADAMTS4) | 5 | 17 | COL1A1 (6), B2M (3), COL3A1 (3), COL1A2 (2), LMAN2 (2), C3 (1), COL5A2 (1), COL6A2 (1), KRT5 (1), SERPINA1 (1), SHRM3 (1) | 1.38 | <0.0001 |
| Matrix metalloproteinase-14 (MMP14) | 11 | 29 | COL1A2 (9), COL1A1 (9), COL3A1 (4), B2M (3), COL5A1 (2), SERPINA1 (2), KRT5 (2), HSPB1 (1), SHRM3 (1), HBB (1), COL23A1 (1), COL5A2 (1), COL4A2 (1), ALB (1), S100A9 (1), COL6A2 (1) | 1.38 | <0.0001 |

↓ ↑ Δ indicate downregulated and upregulated cleaving sites and the nonsurvivor/survivor ratio, respectively. Bracketed numbers indicate the number of cleaved proteins.

**Table S7**

**.** Proteasix Analysis Including Collagen Fragments (Continued)

| Protease (Gene) | N° of Cleaving Sites | | Cleaved Proteins | Δ | *P* |
| --- | --- | --- | --- | --- | --- |
|  | ↓ | ↑ |  |  |  |
| Granzyme M (GZMM) | 0 | 10 | SERPINA1 (3), COL3A1 (2), HSPB1 (1), CRNN (1), B2M (1), APOA1 (1), FBXO31 (1) | 1.35 | <0.0001 |
| Matrix metalloproteinase-25 (MMP25) | 10 | 28 | COL1A1 (7), COL1A2 (6), COL3A1 (4), B2M (3), COL5A1 (2), SERPINA1 (2), KRT5 (2), HSPB1 (1), POTEF (1), SHRM3 (1), APOA1 (1), COL23A1 (1), LMAN2 (1), COL25A1 (1), COL13A1 (1), COL4A2 (1), FBXO31 (1), ALB (1), LEMD3 (1) | 1.35 | <0.0001 |
| Matrilysin (MMP7) | 28 | 59 | COL1A1 (17), SERPINA1 (12), COL1A2 (8), COL3A1 (8), COL5A1 (4), B2M (3), FGA (3), COL5A2 (3), HSPB1 (2), CRNN (2), POTEF (2), COL23A1 (2), UMOD (2), COL25A1 (2), COL13A1 (2), COL4A2 (2), C3 (2), COL6A2 (2), SHRM3 (1), HBB (1), FXYD2 (1), KRT5 (1), LMAN2 (1), FBXO31 (1), ALB (1), S100A9 (1), LEMD3 (1) | 1.35 | <0.0001 |
| Matrix metalloproteinase-9 (MMP9) | 27 | 48 | COL1A1 (19), COL1A2 (10), COL3A1 (8), SERPINA1 (5), COL5A1 (4), COL5A2 (3), HBB (2), COL23A1 (2), COL25A1 (2), COL13A1 (2), ALB (2), C3 (2), B2M (1), POTEF (1), SHRM3 (1), APOA1 (1), UMOD (1), KRT5 (1), LMAN2 (1), FGA (1), COL4A2 (1), FBXO31 (1), MORN4 (1), S100A9 (1), LEMD3 (1), COL6A2 (1) | 1.35 | <0.0001 |
| Stromelysin-1 (MMP3) | 12 | 22 | COL1A1 (12), COL1A2 (5), COL3A1 (3), SERPINA1 (2), B2M (1), HSPB1 (1), CRNN (1), SHRM3 (1), HBB (1), COL23A1 (1), UMOD (1), COL25A1 (1), COL5A2 (1), C3 (1), S100A9 (1), COL6A2 (1) | 1.34 | <0.0001 |
| Calpain-2 catalytic subunit (CAPN2) | 26 | 50 | COL1A1 (19), COL3A1 (8), COL1A2 (7), SERPINA1 (5), B2M (4), COL5A2 (3), COL5A1 (3), HSPB1 (2), COL6A2 (2), KRT5 (2), COL4A2 (2), COL13A1 (2), SHRM3 (2), FGA (2), MORN4 (2), POTEF (1), FBXO31 (1), LEMD3 (1), APOA1 (1), LMAN2 (1), S100A9 (1), ALB (1), C3 (1), HBB (1), COL23A1 (1), UMOD (1) | 1.32 | <0.0001 |

↓ ↑ Δ indicate downregulated and upregulated cleaving sites and the nonsurvivor/survivor ratio, respectively. Bracketed numbers indicate the number of cleaved proteins.

**Table S7**

**.** Proteasix Analysis Including Collagen Fragments (Stops)

| Protease (Gene) | N° of Cleaving Sites | | Cleaved Proteins | Δ | *P* |
| --- | --- | --- | --- | --- | --- |
|  | ↓ | ↑ |  |  |  |
| Calpain-1 catalytic subunit (CAPN1) | 26 | 47 | COL1A1 (19), COL3A1 (8), COL1A2 (7), SERPINA1 (7), B2M (4), COL5A1 (3), COL5A2 (3), COL13A1 (2), COL6A2 (2), FGA (2), HBB (2), HSPB1 (2), KRT5 (2), MORN4 (2), ALB (1), APOA1 (1), C3 (1), FBXO31 (1), LEMD3 (1), S100A9 (1), SHRM3 (1), UMOD (1) | 1.31 | <0.0001 |
| Macrophage metalloelastase (MMP12) | 22 | 30 | COL1A1 (16), COL1A2 (9), COL3A1 (8), COL5A1 (3), COL5A2 (3), SERPINA1 (2), SHRM3 (1), HBB (1), COL23A1 (1), KRT5 (1), LMAN2 (1), COL25A1 (1), COL13A1 (1), COL4A2 (1), FBXO31 (1), S100A9 (1), COL6A2 (1) | 1.3 | <0.0001 |
| Collagenase 3 (MMP13) | 9 | 13 | COL1A1 (7), COL1A2 (6), COL3A1 (3), B2M (1), SHRM3 (1), SERPINA1 (1), COL23A1 (1), COL5A2 (1), COL6A2 (1) | 1.22 | <0.0001 |
| Neprilysin (MME) | 11 | 20 | COL1A1 (7), COL1A2 (4), B2M (3), LMAN2 (2), COL3A1 (2), COL5A2 (2), CRNN (1), SHRM3 (1), SERPINA1 (1), FXYD2 (1), COL23A1 (1), UMOD (1), KRT5 (1), FGA (1), COL13A1 (1), S100A9 (1), COL6A2 (1) | 1.2 | <0.0001 |
| Interstitial collagenase (MMP1) | 6 | 8 | COL1A1 (4), COL1A2 (4), COL3A1 (3), SERPINA1 (1), COL5A1 (1), COL6A2 (1) | 1.2 | 0.0003 |
| Granzyme A (GZMA) | 3 | 10 | COL1A1 (2), KRT5 (2), COL3A1 (2), HSPB1 (1), COL5A1 (1), HBB (1), APOA1 (1), UMOD (1), COL1A2 (1), ALB (1) | 1.14 | 0.0006 |
| Plasminogen (PLG) | 4 | 8 | COL1A1 (2), KRT5 (2), COL3A1 (2), HSPB1 (1), COL5A1 (1), B2M (1), UMOD (1), FGA (1), ALB (1) | 1.08 | 0.0156 |

↓ ↑ Δ indicate downregulated and upregulated cleaving sites and the nonsurvivor/survivor ratio, respectively. Bracketed numbers indicate the number of cleaved proteins.

**Table S8**

**.** Proteasix Analysis Excluding Collagen Fragments (Starts)

| Protease (Gene) | N° of Cleaving Sites | | Cleaved Proteins | Δ | *P* |
| --- | --- | --- | --- | --- | --- |
|  | ↓ | ↑ |  |  |  |
| Cathepsin E (CTSE) | 1 | 21 | SERPINA1 (13), C3 (2), POTEF (1), CRNN (1), MORN4 (1), APOA1 (1), ALB (1), LMAN2 (1), HSPB1 (1) | 1.97 | <0.0001 |
| Cathepsin D (CTSD) | 1 | 22 | SERPINA1 (13), C3 (2), POTEF (1), CRNN (1), MORN4 (1), APOA1 (1), ALB (1), LMAN2 (1), HSPB1 (1), LEMD3 (1) | 1.92 | <0.0001 |
| Matrix metalloproteinase-14 (MMP14) | 0 | 12 | B2M (3), SERPINA1 (2), KRT5 (2), HBB (1), S100A9 (1), ALB (1), HSPB1 (1), SHRM3 (1) | 1.90 | <0.0001 |
| Neutrophil elastase (ELANE) | 1 | 15 | SERPINA1 (4), HBB (2), B2M (2), FBXO31 (2), CRNN (1), APOA1 (1), POTEF (1), HSPB1 (1), LEMD3 (1), KRT5 (1) | 1.89 | <0.0001 |
| Matrix metalloproteinase-25 (MMP25) | 0 | 15 | B2M (3), SERPINA1 (2), KRT5 (2), APOA1 (1), ALB (1), LMAN2 (1), HSPB1 (1), LEMD3 (1), FBXO31 (1), SHRM3 (1), POTEF (1) | 1.89 | <0.0001 |
| Cathepsin G (CTSG) | 1 | 20 | SERPINA1 (11), POTEF (2), CRNN (1), B2M (1), MORN4 (1), APOA1 (1), HSPB1 (1), LEMD3 (1), KRT5 (1), SHRM3 (1) | 1.87 | <0.0001 |
| Cathepsin L1 (CTSL) | 1 | 26 | SERPINA1 (10), B2M (4), HBB (2), CRNN (1), S100A9 (1), MORN4 (1), APOA1 (1), ALB (1), LMAN2 (1), HSPB1 (1), C3 (1), KRT5 (1), FBXO31 (1), SHRM3 (1) | 1.85 | 0.0001 |
| Cathepsin B (CTSB) | 1 | 16 | SERPINA1 (4), HBB (2), KRT5 (2), CRNN (1), S100A9 (1), MORN4 (1), APOA1 (1), ALB (1), LMAN2 (1), HSPB1 (1), C3 (1), SHRM3 (1) | 1.82 | <0.0001 |
| Cathepsin S (CTSS) | 4 | 28 | SERPINA1 (10), B2M (3), HBB (2), MORN4 (2), FGA (2), KRT5 (2), FBXO31 (2), CRNN (1), S100A9 (1), APOA1 (1), ALB (1), LMAN2 (1), HSPB1 (1), C3 (1), SHRM3 (1), POTEF (1) | 1.79 | 0.0002 |
| Meprin A subunit alpha (MEP1A) | 5 | 29 | SERPINA1 (11), B2M (3), FGA (3), CRNN (2), HBB (2), ALB (2), C3 (2), KRT5 (2), S100A9 (1), MORN4 (1), APOA1 (1), UMOD (1), HSPB1 (1), FBXO31 (1), SHRM3 (1) | 1.61 | 0.0025 |

↓ ↑ Δ indicate downregulated and upregulated cleaving sites and the nonsurvivor/survivor ratio, respectively. Bracketed numbers indicate the number of cleaved proteins.

**Table S8**

**.** Proteasix Analysis Excluding Collagen Fragments (Stops)

| Protease (Gene) | N° of Cleaving Sites | | Cleaved Proteins | Δ | *P* |
| --- | --- | --- | --- | --- | --- |
|  | ↓ | ↑ |  |  |  |
| Matrix metalloproteinase-9 (MMP9) | 3 | 20 | SERPINA1 (5), HBB (2), ALB (2), C3 (2), B2M (1), S100A9 (1), MORN4 (1), APOA1 (1), FGA (1), UMOD (1), POTEF (1), LMAN2 (1), LEMD3 (1), KRT5 (1), FBXO31 (1), SHRM3 (1) | 1.61 | 0.0001 |
| Cathepsin K (CTSK) | 4 | 21 | SERPINA1 (6), HBB (2), B2M (2), FGA (2), HSPB1 (2), CRNN (1), S100A9 (1), MORN4 (1), APOA1 (1), ALB (1), UMOD (1), LMAN2 (1), KRT5 (1), FBXO31 (1), SHRM3 (1), POTEF (1) | 1.60 | 0.0028 |
| Matrilysin (MMP7) | 6 | 31 | SERPINA1 (12), B2M (3), FGA (3), CRNN (2), UMOD (2), POTEF (2), HSPB1 (2), C3 (2), HBB (1), S100A9 (1), ALB (1), LMAN2 (1), LEMD3 (1), KRT5 (1), FXYD2 (1), FBXO31 (1), SHRM3 (1) | 1.51 | 0.0036 |
| Calpain-2 catalytic subunit (CAPN2) | 5 | 24 | SERPINA1 (5), B2M (4), MORN4 (2), FGA (2), HSPB1 (2), KRT5 (2), SHRM3 (2), HBB (1), S100A9 (1), APOA1 (1), ALB (1), UMOD (1), POTEF (1), LMAN2 (1), C3 (1), LEMD3 (1), FBXO31 (1) | 1.49 | 0.0007 |
| Calpain-1 catalytic subunit (CAPN1) | 5 | 24 | SERPINA1 (7), B2M (4), HBB (2), MORN4 (2), FGA (2), HSPB1 (2), KRT5 (2), S100A9 (1), APOA1 (1), ALB (1), UMOD (1), C3 (1), LEMD3 (1), FBXO31 (1), SHRM3 (1) | 1.48 | 0.0025 |
| Stromelysin-1 (MMP3) | 1 | 9 | SERPINA1 (2), CRNN (1), HBB (1), B2M (1), S100A9 (1), UMOD (1), HSPB1 (1), C3 (1), SHRM3 (1) | 1.42 | 0.0082 |
| Neprilysin (MME) | 3 | 10 | B2M (3), LMAN2 (2), CRNN (1), S100A9 (1), FGA (1), SERPINA1 (1), UMOD (1), KRT5 (1), FXYD2 (1), SHRM3 (1) | 1.11 | 0.043 |

↓ ↑ Δ indicate downregulated and upregulated cleaving sites and the nonsurvivor/survivor ratio, respectively. Bracketed numbers indicate the number of cleaved proteins.


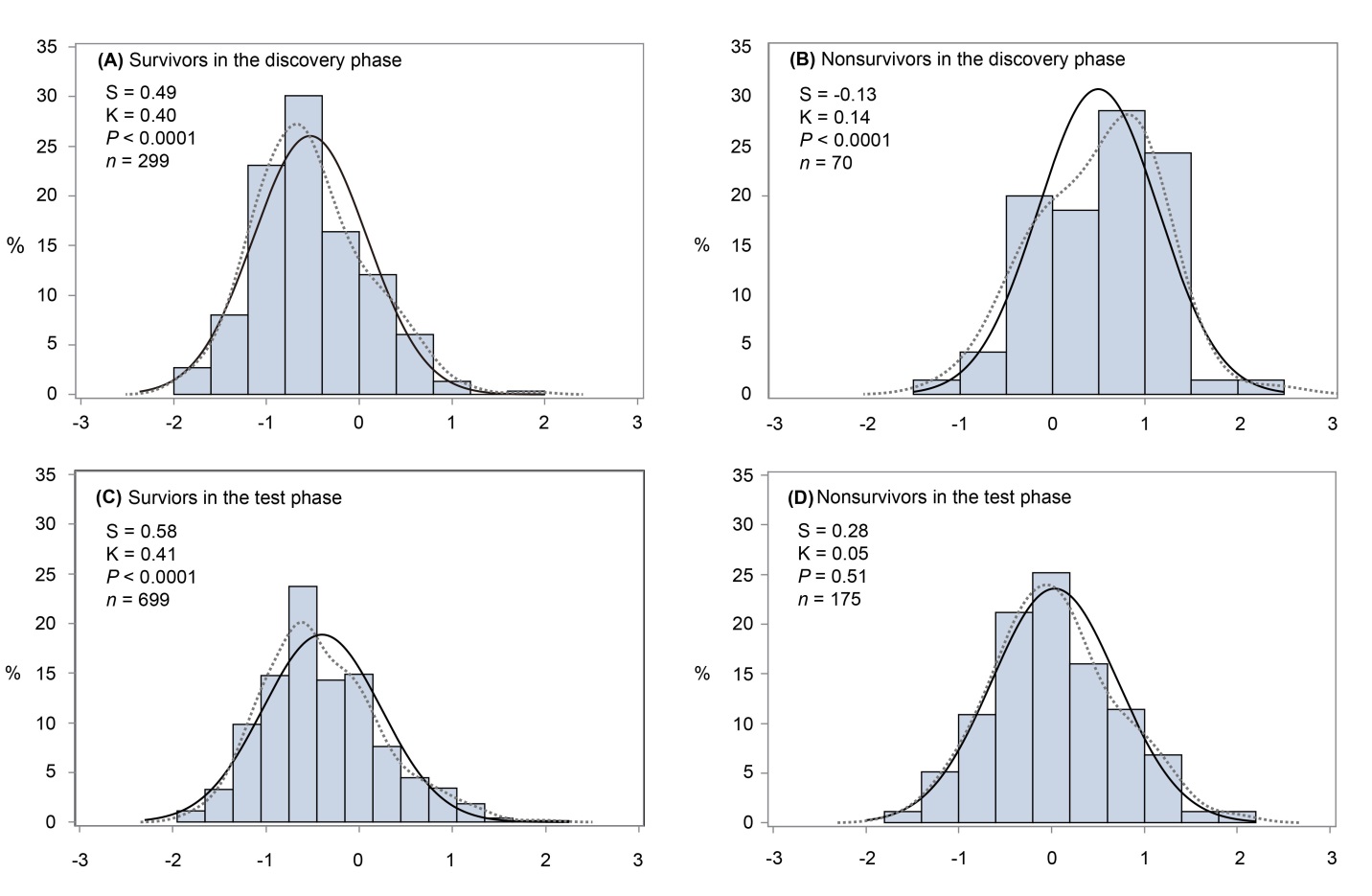


**Figure S1**

Distributions of multidimensional urinary ACM128 in survivors (A, C) and nonsurvivors (B, D) in the discovery and test datasets. N, M, S and K indicate the number of patients, the mean and the coefficients of skewness and kurtosis. The solid and dotted lines represent the normal and kernel density distributions. The *P*values are for departure of the actually observed distribution from normality according to the Shapiro-Wilk statistic.


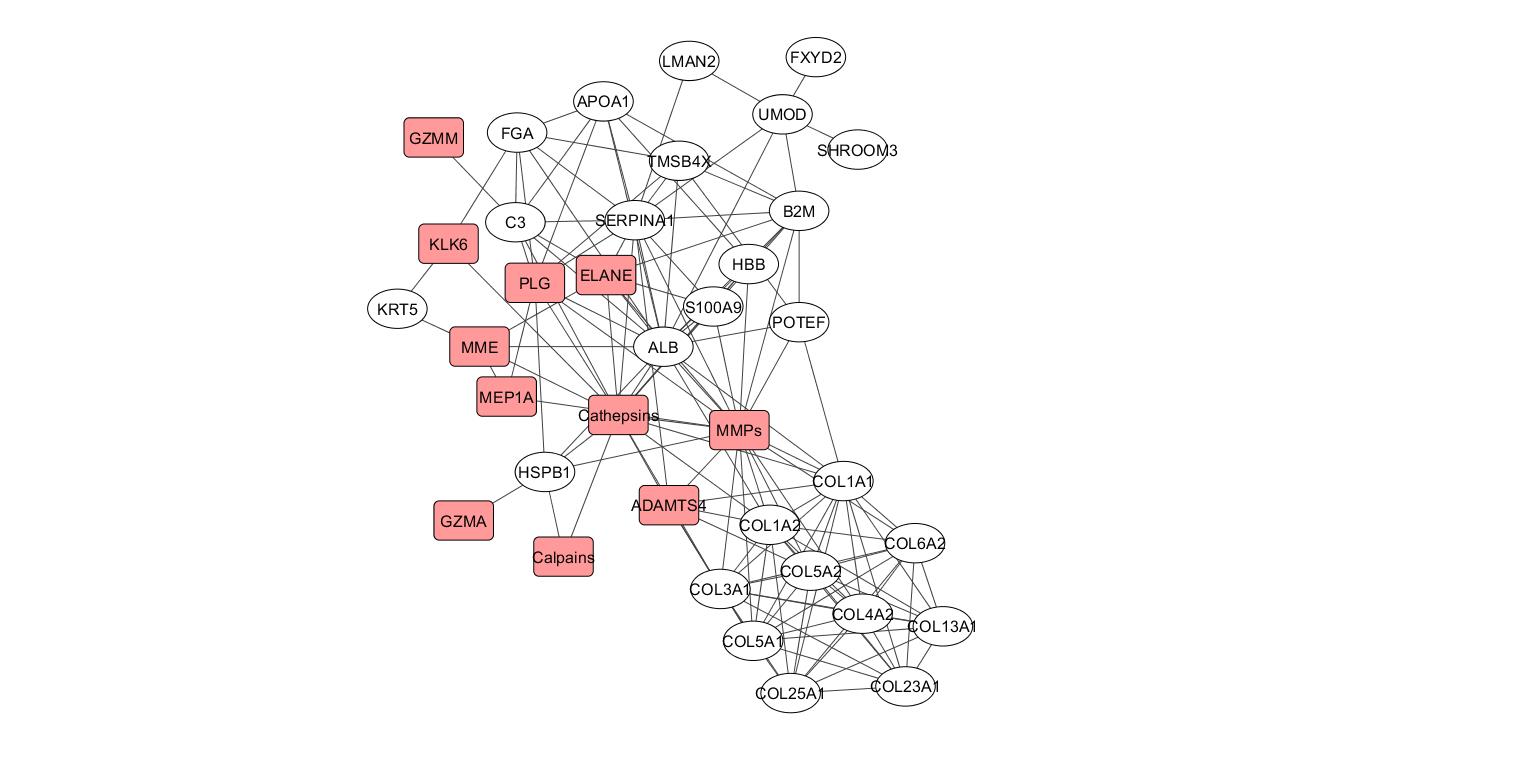


**Figure S2**

Protein-protein interactome derived from 30 proteins corresponding to 63 sequenced urinary peptides included in the ACM128 classifier predictive of the 1-year mortality post-ICU mortality and the in-silico predicted 27 proteases generating the peptide fragments (Table E6). Collagen fragments were included in the analysis. The network included 57 nodes and 295 edges, with a protein-protein interaction *P* of 10‑16. Nodes disconnected from the network were not displayed. Nodes in red color indicate proteases predicted in silico (Table E6). Proteases belonging to the same family were displayed as a group in the network: matrix metalloproteinases (MMPs; i.e., MMP1, MMP2, MMP3, MMP7, MMP8, MMP9, MMP12, MMP13, MMP14 and MMP25); cathepsins (i.e., cathepsins B, D, E, G, K, L1 and S); granzyme (i.e., granzymes A and M) and calpains (i.e., the calpain 1 and 2 catalytic subunits).


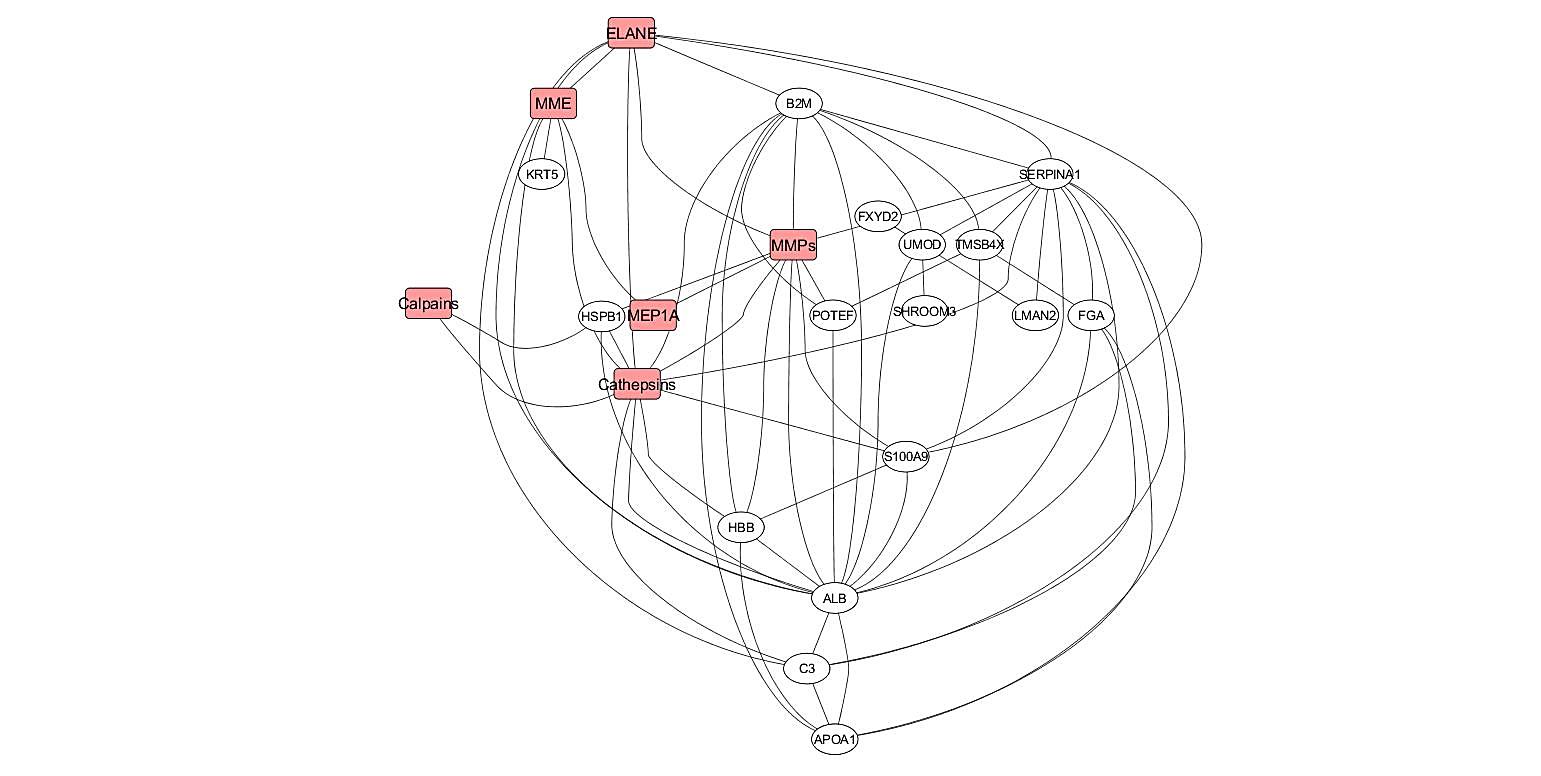


**Figure S3**

Protein-protein interactome derived from 20 proteins corresponding to 35 sequenced urinary peptides included in the ACM128 classifier predictive of the 1-year mortality post ICU mortality and the in-silico predicted 17 proteases generating the peptide fragments (Table E7). Twenty-eight collagen fragments were excluded from analysis. The network included 37 nodes and 122 edges, with a protein-protein interaction *P* of 10‑16. Nodes disconnected from the network were not displayed. Nodes in red color indicate proteases predicted in silico (Table E7). Proteases belonging to the same family were displayed as a group in the network: matrix metalloproteinases (MMPs; i.e., MMP3, MMP7, MMP9, MMP14 and MMP25); cathepsins (i.e., cathepsin B, D, E, G, K, L1 and S); granzymes (i.e., granzyme A and M) and calpains (i.e., the calpain 1 and 2 catalytic subunits).
